# Lysine butyrylation of HSP90 regulated by KAT8 and HDAC11 confers chemoresistance

**DOI:** 10.1038/s41421-023-00570-y

**Published:** 2023-07-18

**Authors:** Yan He, Can-Can Zheng, Jing Yang, Shu-Jun Li, Tao-Yang Xu, Xian Wei, Wen-You Chen, Zhi-Li Jiang, Jiao-Jiao Xu, Guo-Geng Zhang, Chao Cheng, Kui-Sheng Chen, Xing-Yuan Shi, Da-Jiang Qin, Jin-Bao Liu, Bin Li

**Affiliations:** 1 Key Laboratory of Biological Targeting Diagnosis, Therapy and Rehabilitation of Guangdong Higher Education Institutes, The Fifth Affiliated Hospital of Guangzhou Medical University, Guangzhou, Guangdong China; 2 MOE Key Laboratory of Tumor Molecular Biology, National Engineering Research Center of Genetic Medicine, College of Life Science and Technology, Jinan University, Guangzhou, Guangdong China; 3 Department of Thoracic Surgery, The First Affiliated Hospital of Jinan University, Guangzhou, Guangdong China; 4 Department of Radiation Oncology, The Fifth Affiliated Hospital of Guangzhou Medical University, Guangzhou, Guangdong China; 5 Department of Thoracic Surgery, Sun Yat-sen University First Affiliated Hospital, Guangzhou, Guangdong China; 6 Department of Pathology, The First Affiliated Hospital of Zhengzhou University, Henan Key Laboratory of Tumor Pathology, Zhengzhou, Henan China; 7 Key Laboratory of Protein Modification and Degradation, State Key Laboratory of Respiratory Disease, School of Basic Medical Sciences, Guangzhou Medical University, Guangzhou, Guangdong China

**Keywords:** Cancer therapeutic resistance, Post-translational modifications, Proteomic analysis, Drug development

## Abstract

Posttranslational modification dramatically enhances protein complexity, but the function and precise mechanism of novel lysine acylation modifications remain unknown. Chemoresistance remains a daunting challenge to successful treatment. We found that lysine butyrylation (Kbu) is specifically upregulated in chemoresistant tumor cells and tissues. By integrating butyrylome profiling and gain/loss-of-function experiments, lysine 754 in HSP90 (HSP90 K754) was identified as a substrate for Kbu. Kbu modification leads to overexpression of HSP90 in esophageal squamous cell carcinoma (ESCC) and its further increase in relapse samples. Upregulation of HSP90 contributes to 5-FU resistance and can predict poor prognosis in cancer patients. Mechanistically, HSP90 K754 is regulated by the cooperation of KAT8 and HDAC11 as the writer and eraser, respectively; SDCBP increases the Kbu level and stability of HSP90 by binding competitively to HDAC11. Furthermore, SDCBP blockade with the lead compound V020-9974 can target HSP90 K754 to overcome 5-FU resistance, constituting a potential therapeutic strategy.

## Introduction

Despite significant advances in surgery and chemotherapy, drug resistance persists as a barrier to therapeutic success in cancer and accounts for the high mortality of this disease^1,2^. Gene mutations have been found in certain signaling pathways involved in DNA repair, membrane transport, autophagy and mitochondrial dysfunction^3–5^, but genomic alterations cannot completely explain the complex mechanisms underlying chemoresistance. Posttranslational modification (PTM) increases the functional diversity of proteins in physiological and pathological processes^6^. Recent breakthroughs in high-resolution mass spectrometry (MS)-based proteomics have paved the way for identifying novel PTMs in various organisms^7^, but little is known about the biological functions of the novel lysine (K) acylation modifications.

Lysine butyrylation (Kbu) is a novel PTM that is found in histone and nonhistone proteins^8^. Kbu has been identified in fungi, plants and animals^9–11^, but a global landscape of nonhistone protein Kbu modification in humans is lacking. More importantly, the writer and eraser responsible for Kbu deposition and removal in humans, respectively, have not been reported. In this study, among the newly identified lysine acylation modifications examined, the level of only Kbu was significantly changed in fluorouracil (5-FU)-resistant (FR) esophageal squamous cell carcinoma (ESCC) cells compared with the parental cells, thus became our research focus. Here, our quantitative analysis of the butyrylome of 5-FU-resistant ESCC cells, as well as functional studies, showed that Kbu modification of lysine 754 (K754) in heat shock protein 90 (HSP90), a highly conserved molecular chaperone involved in signal transduction and protein degradation, contributes significantly to chemoresistance. The acylase and deacylase responsible for HSP90 Kbu were also investigated.

In this study, we aimed to reveal the biological and clinical significance of Kbu modification in cancer and to screen and validate lysine acetyltransferase 8 (KAT8) and histone deacetylase 11 (HDAC11) as writer and eraser of Kbu. We also determined whether Kbu modification of HSP90 is regulated by syndecan binding protein (SDCBP) via competitive protein binding. Furthermore, the results of pharmacological inhibition of HSP90 Kbu with a lead compound suggest a potential therapeutic strategy to overcome chemoresistance.

## Results

### Global landscape of Kbu modification in human cancer

**Fig. 1:**
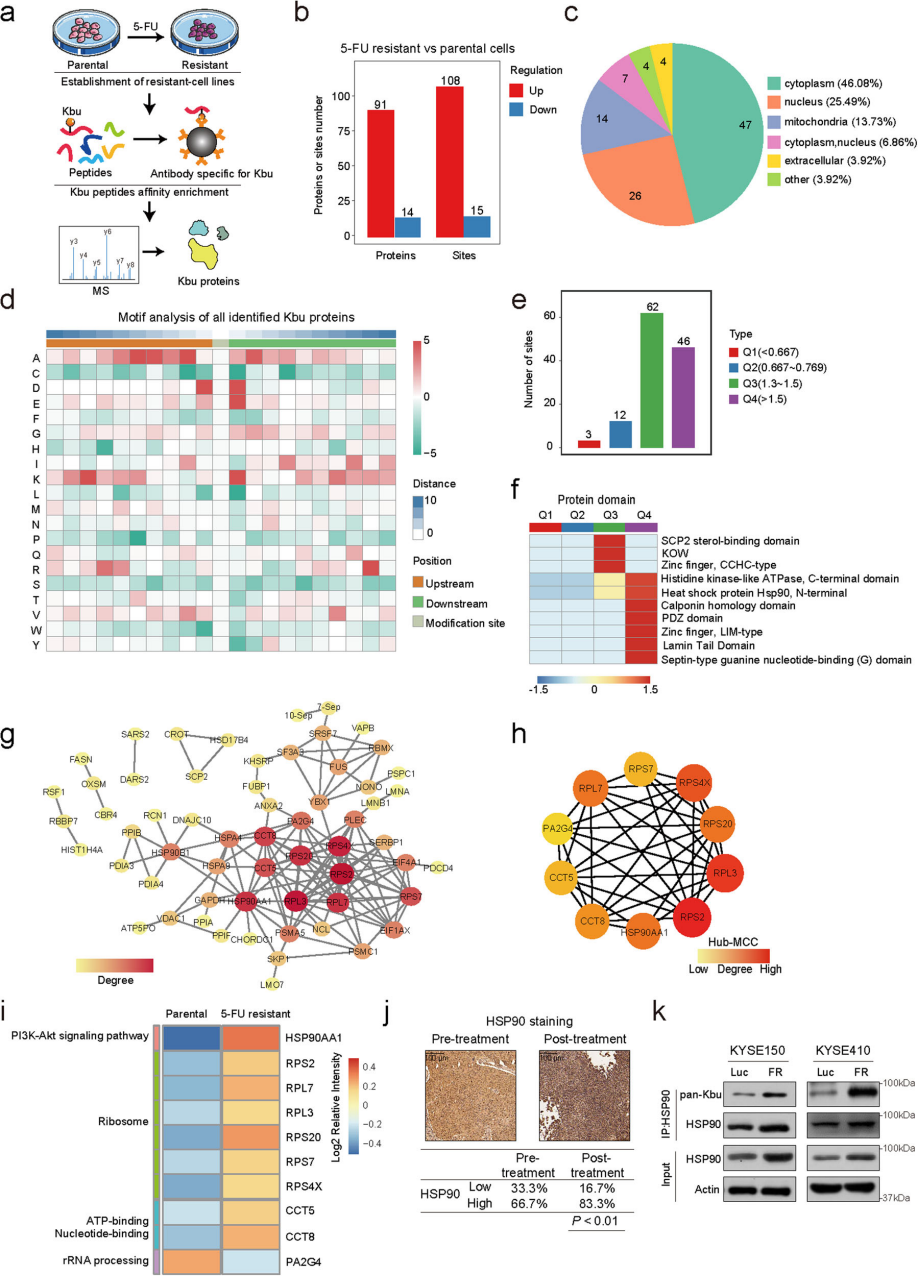
Lysine butyrylome profile in cancer chemoresistance and the important role of Kbu modification of HSP90. **a** Scheme of the lysine butyrylome in 5-FU-resistant cell line KYSE150-FR and the parental cells KYSE150. **b** DEKPs and DEKSs identified by butyrylome analysis of resistant and parental cells. **c** Subcellular distribution of DEKPs in 5-FU-resistant and parental cells. **d** Heatmap indicating the enrichment (red) and depletion (green) of amino acids at each position flanking the Kbu sites. **e** The DEKSs were classified into four clusters by the Q classification method for further enrichment analysis. **f** GO enrichment analysis showing the enriched protein domains of DEKPs. **g** PPI network showing the relationships among the DEKPs. **h** Top 10 hub proteins in the PPI network. **i** Enrichment analysis of DEKPs identified by butyrylome analysis revealed the important role of the HSP90AA1-PI3K/AKT pathway in chemoresistance in cancer. **j** HSP90 expression levels in paired ESCC biopsy and post-5-FU-treatment relapse surgical specimens. **k** Kbu modification of HSP90 was increased in 5-FU-resistant cells.

To investigate the impact of newly discovered lysine acylation modifications on chemoresistance in cancer, nine lysine acylation pan-antibodies, as well as dimethyllysine and trimethyllysine pan-antibodies, were used to compare our previously established paired 5-FU-sensitive/resistant ESCC cells^12^. We noted an obvious increase only in Kbu but not other lysine acylation modifications in 5-FU-resistant ESCC cells (Supplementary Fig. S1). The functional role of Kbu in cancer is currently unknown. To map the butyrylome of 5-FU-resistant cells, Kbu pan-antibody-conjugated beads were used to enrich the modified peptides prior to high-resolution liquid chromatography–tandem MS (LC–MS/MS) analysis (Fig. 1a). Altogether, we identified 123 differentially expressed Kbu sites (DEKSs) in 105 differentially expressed Kbu proteins (DEKPs) in 5-FU-resistant cells compared with the parental cells (Fig. 1b; Supplementary Dataset S1). Subcellular localization analysis revealed that nearly half (46.08%) of the DEKPs were localized in the cytoplasm (Fig. 1c). We next examined the amino acids flanking the identified Kbu sites, and enrichment of negatively charged amino acid was found at −1 and +1 positions of Kbu sites (Fig. 1d). Furthermore, four distinct butyrylome subgroups (Q1, Q2, Q3 and Q4) were defined by cluster analysis of the DEKSs (Fig. 1e). Q1 (< 0.667) and Q2 (0.667–0.769) subgroups include the modification sites with decreased Kbu levels, while Q3 (1.3–1.5) and Q4 (> 1.5) subgroups include the modification sites with increased Kbu levels. Gene Ontology (GO) enrichment analysis suggested the possible functions of the DEKPs in chemoresistance (Supplementary Fig. S2a–c). In addition, protein domain enrichment analysis revealed that the DEKPs contained many functional domains, such as the N-terminal of HSP90 (Fig. 1f).

### Proteome analysis suggests that Kbu modification of HSP90 is essential for cancer chemoresistance

Centrality analysis of the DEKPs in chemoresistance was conducted to identify the hub genes in the protein–protein interaction (PPI) network based on the STRING database (Fig. 1g). The 10 proteins with the highest linkage degrees in the network diagram were then identified using the MCC algorithm (Fig. 1h). More importantly, GO enrichment analysis suggested that HSP90 AA1 (referred to as HSP90 throughout the manuscript) with the most significant increase in Kbu modifications participates in the regulation of the PI3K/AKT pathway, a key signaling pathway in modulating 5-FU resistance, as reported by us and others^12,13^ (Fig. 1i). Therefore, HSP90 was selected as our research focus. To examine the clinical relevance of HSP90 in ESCC chemoresistance, we collected paired biopsy and surgical ESCC specimens before and after 5-FU-based neoadjuvant therapy. We found that HSP90 expression was markedly higher in the relapse samples, further supporting the involvement of HSP90 in chemoresistance (Fig. 1j). Furthermore, Kbu modification of HSP90 was confirmed to be increased in 5-FU-resistant ESCC cells (Fig. 1k), suggesting its potential role in chemoresistance.

### Kbu modification of HSP90 K754 leads to chemoresistance in cancer

**Fig. 2:**
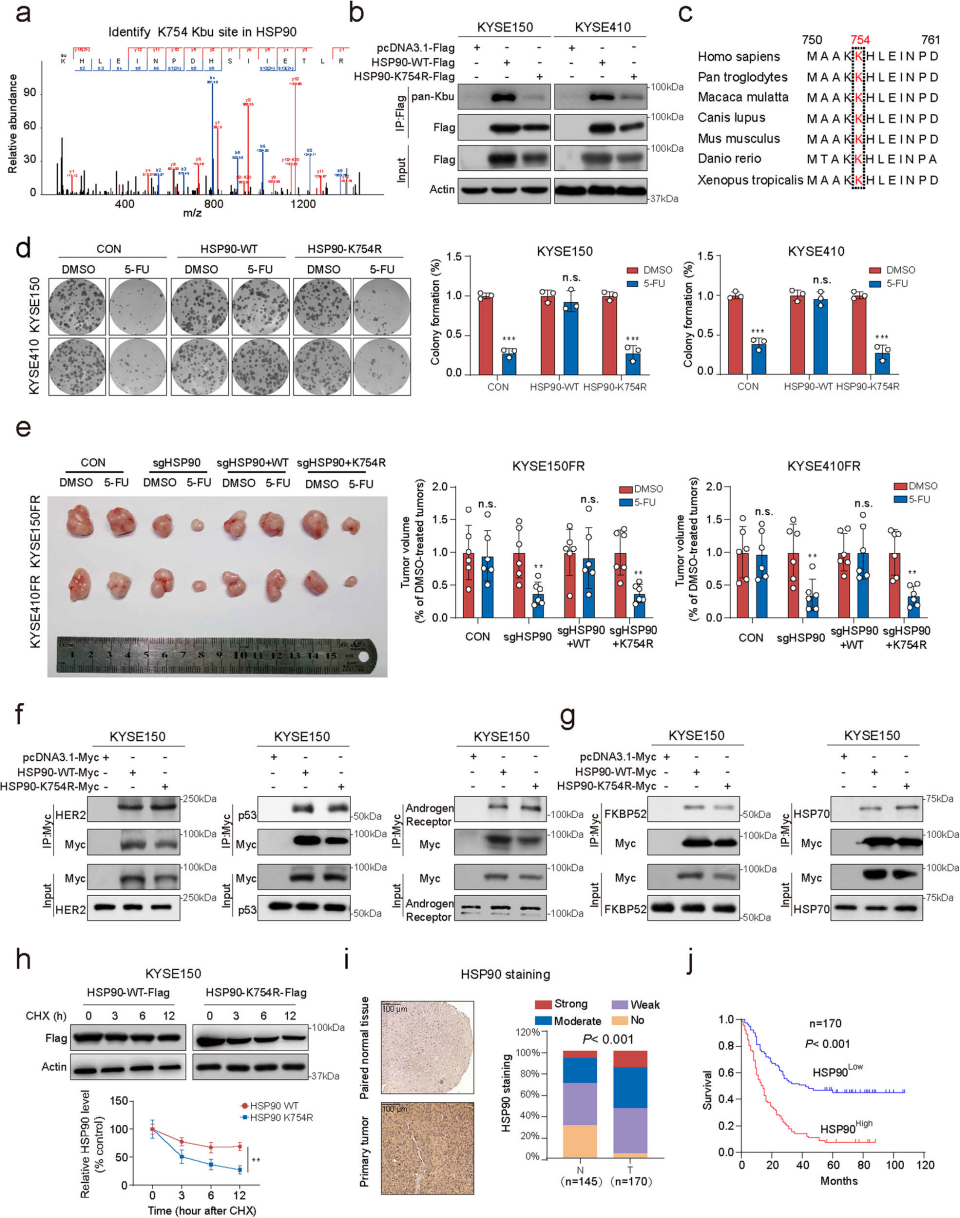
Butyrylation of HSP90 at K754 is essential for cancer chemoresistance. **a** The MS/MS spectrum of the modified HSP90 peptide. **b** KYSE150 and KYSE410 were transiently transfected with the pcDNA3.1-Flag plasmid expressing wild-type HSP-90 (HSP90-WT) or the HSP90-K754R mutant and were then subjected to immunoprecipitation/immunoblotting (IB) with the indicated antibodies. **c** The sequences surrounding K754 in HSP90 among seven species were aligned. Lysine 754 of HSP90 was colored in red. **d** KYSE150 and KYSE410 were transiently transfected with the pcDNA3.1-Flag plasmid expressing HSP90-WT or the HSP90-K754R mutant. Colony formation assay showing that the HSP90 K754R mutation abolished the promoting effect of HSP90-WT on 5-FU resistance in ESCC cells. **e** Representative images and quantitative analyses of tumor xenografts established with ESCC cells as indicated in the presence or absence of 5-FU. HSP90-WT or the HSP90-K754R mutant was re-overexpressed in HSP90-deficient ESCC cells. **f**, **g** The effect of HSP90 K754R mutation on its interaction with client proteins and cochaperones. **h** A CHX (100 μg/mL) chase assay was used to compare the stability of the wild-type HSP90 (HSP90-WT) and HSP90-K754R mutant proteins in ESCC cells. **i** Representative images and expression pattern of HSP90 in 170 ESCC and 145 paired adjacent normal tissues. **j** Kaplan–Meier analysis of overall survival for 170 ESCC patients stratified by the tumor HSP90 level. Bars, SDs; *P < 0.05; **P < 0.01; ***P < 0.001.

We next explored the Kbu sites in HSP90 that function in mediating chemoresistance. Among the four Kbu sites in HSP90 identified by butyrylome analysis, mutation (lysine to arginine, mimicking the de-butyrylation state) of only K754 but not the other three sites markedly abolished the effect of wild-type HSP90 on promoting chemoresistance (Supplementary Fig. S2d). K754 Kbu modification of HSP90 was not only indicated by LC–MS/MS analysis (Fig. 2a) but also validated by immunoprecipitation data showing that mutation of K754 to R754 resulted in a significant decrease in the Kbu level (Fig. 2b). Conservation analysis of HSP90 indicated that K754 is a highly conserved site from *Xenopus tropicalis* to *Homo sapiens* (Fig. 2c). Results from a series of gain/loss-of-function experiments indicated that HSP90 promoted 5-FU resistance of ESCC cells in vitro and in vivo and that this effect was diminished when K754 was mutated (Fig. 2d, e; Supplementary Fig. S2e). Collectively, these data demonstrate that K754 is the major functional Kbu site in HSP90 that mediates drug resistance.

HSP90 is a molecular chaperone that participates in stabilizing and activating many proteins in cells. To study the impact of K754 butyrylation status on HSP90 function, we performed a series of experiments. As shown in Fig. 2f, g, the K754R mutant did not affect the interaction between HSP90 with client proteins^14–16^ (HER2, p53 and Androgen Receptor) and cochaperones^17,18^ (FKBP52 and HSP70). However, the results of our cycloheximide (CHX) chase assay indicated that mutation of K754 dramatically impaired HSP90 protein stability, suggesting the important role of Kbu modification in the upregulation of HSP90 in cancer (Fig. 2h). These data suggest that Kbu at 754 is important for HSP90 stability. We next explored the clinical relevance of HSP90 in a cohort of 170 ESCC tissues and 145 matched adjacent normal tissues. Immunohistochemical (IHC) staining showed that the expression level of HSP90 in tumor tissues (53.52%, 91/170) was significantly higher than that in adjacent normal tissues (30.34%, 44/145) (*P* < 0.001, Fig. 2i), and high HSP90 expression was associated with advanced pathological T stage and tumor stage (*P* < 0.001, Supplementary Table S1). Kaplan–Meier survival analysis indicated that patients with high HSP90 levels had markedly shorter survival times (median survival time = 14.0 months) than patients with low HSP90 levels (median survival time = 42.0 months) (log-rank test, *P* < 0.001; Fig. 2j).

### KAT8 and HDAC11 are writer and eraser for Kbu modification of HSP90, respectively

**Fig. 3:**
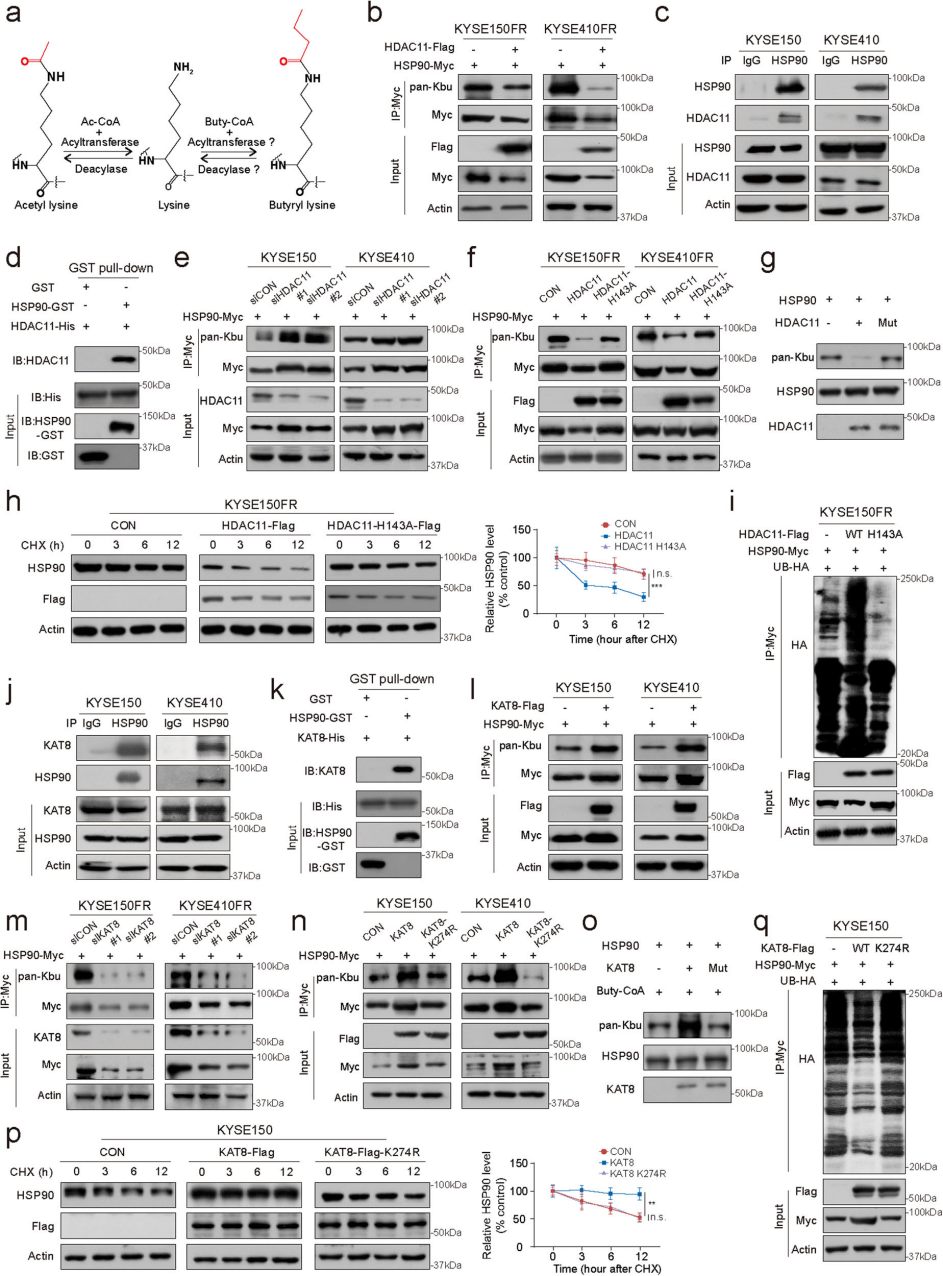
HDAC11 and KAT8 are the eraser and writer enzymes for Kbu modification of HSP90. **a** Diagram of enzymatic reaction for acetyl-lysine and the hypothesized mechanism for butyryllysine. **b** HDAC11 decreased Kbu modification of HSP90. **c** The interaction between endogenous HDAC11 and HSP90 was investigated by immunoprecipitation. **d** Purified GST (lane 1) or HSP90-GST recombinant protein (lane 2) was immobilized on Glutathione-Sepharose beads and incubated with HDAC11-His recombinant protein, followed by immunoblotting. **e** Knockdown of HDAC11 increased Kbu modification of HSP90. **f** Effect of the HDAC11-H143A mutant on the HSP90 Kbu level. **g** In vitro deacylase assay was used to determine the Kbu levels of HSP90 in the presence of wild-type HDAC11 or the catalytically inactive mutant of HDAC11 (HDAC11-H143A). **h** Quantification of protein stability after CHX treatment in ESCC cells transfected with wild-type HDAC11 or the HDAC11-H143A mutant. **i** Effect of HDAC11 and HDAC11-H143A on the ubiquitination of HSP90. **j** An immunoprecipitation assay was performed to confirm the endogenous interaction between HSP90 and KAT8. **k** Purified GST (lane 1) or HSP90-GST recombinant protein (lane 2) was immobilized on Glutathione-Sepharose beads and incubated with KAT8-His recombinant protein, followed by immunoblotting. **l** KAT8 increased Kbu modification of HSP90. Effect of KAT8 knockdown (**m**) and KAT8-K274R mutant (**n**) on HSP90 Kbu level. **o** In vitro butyrylation of HSP90. HSP90 was incubated with the purified KAT8 or KAT8-K274R in the presence of buty-CoA as indicated. **p** Quantification of protein stability after CHX treatment in ESCC cells transfected with wild-type KAT8 or HAT-dead KAT8-K274R mutant. **q** Effect of KAT8 and KAT8-K274R on the ubiquitination of HSP90. Bars, SDs; *P < 0.05; **P < 0.01; ***P < 0.001.

Identification of the enzymes responsible for protein acylation and deacylation related to Kbu modification is important but has not been reported. Prompted by the evidence that several of the newly identified PTMs are regulated by enzymes previously classified as histone acetyltransferases (HATs) and histone deacetylases (HDACs)^19^, we performed immunoprecipitation to detect associations between HSP90 and HATs or HDACs (Fig. 3a). Among a panel of proteins in the HDAC superfamily, including HDAC 1–11 and sirtuin (SIRT) 1–7, we found that HDAC1, HDAC3, HDAC11 and SIRT4 can directly interact with HSP90 (Supplementary Fig. S3a), and further experiments showed that only HDAC11 but not the other HDACs could modulate the HSP90 butyrylation status (Supplementary Fig. S3b, c; Fig. 3b). Besides, the binding of HDAC11 to HSP90 was confirmed in ESCC cells by coimmunoprecipitation (co-IP) and glutathione S-transferase (GST) pull-down assay (Fig. 3c, d). Knockdown of HDAC11 enhanced the level of HSP90 Kbu in ESCC cells (Fig. 3e). In contrast, the catalytically inactive mutant of HDAC11 (HDAC11-H143A)^20,21^ lost the ability to regulate the HSP90 Kbu level (Fig. 3f). Consistently, Kbu products were reduced in response to the addition of wild-type HDAC11, but not the mutant, indicating that HDAC11 is a Kbu deacylase in vitro (Fig. 3g). We also found that the stability of HSP90 protein was reduced by ectopic expression of wild-type HDAC11, but not the HDAC11-H143A mutant (Fig. 3h, i). As shown in Supplementary Fig. S3f (left panel), overexpression of HDAC11 decreased the stability of wild-type HSP90 and did not exert a similar effect on the mutant (HSP90-K754R). The above findings show that HDAC11 is a deacylase capable of catalyzing the removal of HSP90 Kbu.

On the other hand, to identify the HATs responsible for HSP90 Kbu modification, the association of a series of HATs with HSP90 was tested by immunoprecipitation. The results showed that only KAT8 can directly interact with HSP90 (Supplementary Fig. S3d), which was further confirmed by the endogenous co-IP and GST pull-down assay (Fig. 3j, k). We found that KAT8 increased the HSP90 butyrylation level (Supplementary Fig. S3e; Fig. 3l), and knockdown of KAT8 decreased HSP90 Kbu (Fig. 3m). Moreover, the catalytically inactive mutant of KAT8 (KAT8-K274R)^22,23^ lost the ability to regulate the HSP90 Kbu status (Fig. 3n). Our in vitro modification reaction data indicated that Kbu level of wild-type HSP90 was induced by KAT8, validating that KAT8 directly acylated HSP90 at K754 (Fig. 3o). The CHX chase assay indicated that KAT8 markedly enhanced the stability of HSP90 protein, and this effect was abolished when the K274 in KAT8 was mutated (Fig. 3p, q). Furthermore, overexpression of KAT8 increased the stability of wild-type HSP90 and did not exhibit a similar effect on the HSP90-K754R mutant (Supplementary Fig. S3f, right panel). Together, we conclude that KAT8 is a writer of HSP90 Kbu modification.

### Identification of SDCBP as a driver of HSP90 Kbu

**Fig. 4:**
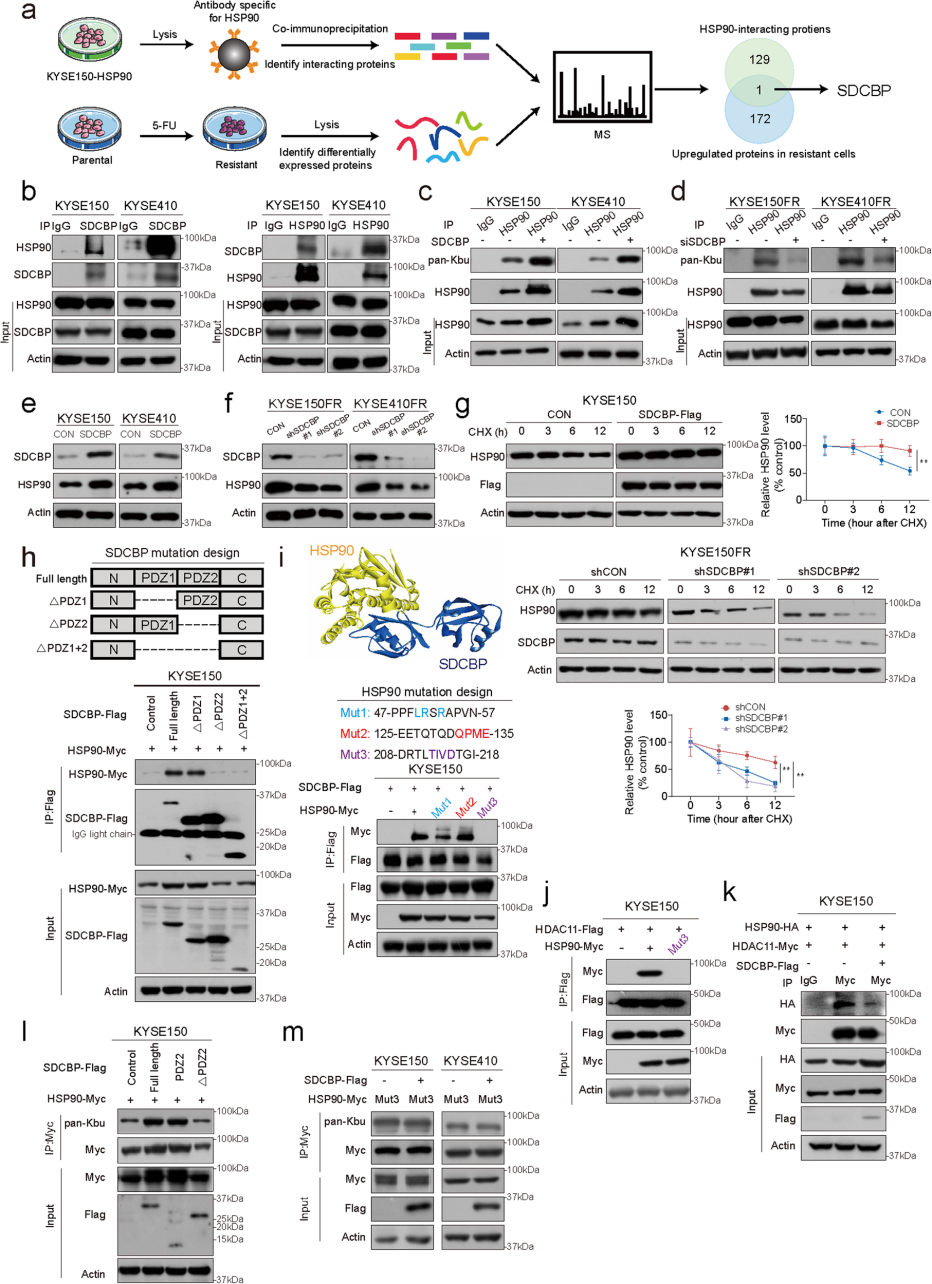
SDCBP is a key regulator of HSP90 Kbu modification. **a** Diagram showing the strategy used to screen for the candidate proteins that are not only upregulated in 5-FU-resistant cells but also interact with HSP90. IP-MS was performed in KYSE150 cells with HSP90 overexpression. Systematic proteomic analysis was conducted in the 5-FU-resistant cell line KYSE150-FR and the parental cells KYSE150. **b** The interaction between endogenous SDCBP and HSP90 was determined by immunoprecipitation. **c** HSP90 Kbu levels were determined in SDCBP-overexpressing ESCC cells. **d** SDCBP knockdown decreased the HSP90 Kbu level. Detection of HSP90 expression in SDCBP-overexpressing (**e**) and SDCBP-knockdown ESCC cells (**f**). **g** Analysis of HSP90 stability in SDCBP-overexpressing and SDCBP-knockdown ESCC cells by a CHX chase assay. **h** Upper panel, schematic diagram of different SDCBP truncation mutant constructs; lower panel, ESCC cells cotransfected with Flag-tagged SDCBP mutants as indicated and Myc-tagged HSP90 were collected for immunoprecipitation. **i** Upper panel, in silico docking simulation showing the interaction between SDCBP and HSP90; middle panel, schematic of the HSP90 mutant constructs; lower panel, cells cotransfected with Flag-tagged SDCBP and Myc-tagged HSP90 mutants were subjected to immunoprecipitation. **j** Co-IP results showing the binding of HDAC11 to wild-type or mutant HSP90. **k** The interactions among HSP90, SDCBP and HDAC11 were determined by co-IP. **l** Comparison of the HSP90 Kbu levels in ESCC cells overexpressing SDCBP-WT, SDCBP-PDZ2, SDCBP-△PDZ2 or vector control. **m** HSP90 Kbu levels were determined in ESCC cells transfected with indicated plasmids. Bars, SDs; *P < 0.05; **P < 0.01; ***P < 0.001.

Further experiments showed that KAT8 expression differed between resistant and parental cells, but HDAC11 expression did not (Supplementary Fig. S3g), prompting us to hypothesize that other proteins cooperate with HDAC11 to regulate the Kbu modification and protein stability of HSP90. On the one hand, we used MS to analyze the differentially expressed proteins (DEPs) in paired 5-FU-sensitive/resistant ESCC cells. Proteomics data identified a total of 310 DEPs, including 172 upregulated and 138 downregulated proteins (Supplementary Fig. S4a and Dataset S2). The DEPs were mainly enriched in the nucleus and cytoplasm (Supplementary Fig. S4b). On the other hand, we performed immunoprecipitation coupled with liquid chromatography–tandem mass spectrometry (IP–MS) to identify the binding partners of HSP90. After overlapping the 172 upregulated proteins with the 129 interacting partners of HSP90 identified by IP-MS (Supplementary Dataset S3), we obtained only 1 candidate protein, SDCBP (Fig. 4a). SDCBP is an adapter protein containing two tandem PDZ domains^24^. Overexpression of SDCBP was observed in various human cancers, including melanoma^25^, breast cancer^26^ and gastric cancer^27^, and plays an important role in cancer progression. SDCBP was selected as the protein of interest since it (1) was confirmed to be upregulated in both resistant cell lines (Supplementary Fig. S4c), (2) bound to both endogenous and exogenous HSP90, and vice versa (Fig. 4b; Supplementary Fig. S4d), (3) was capable of regulating Kbu modification of HSP90 (Fig. 4c, d). Several hundred proteins have been identified as the clients of HSP90, inspiring us to detect whether SDCBP could act as a client of HSP90. CHX and western blot assays showed that HSP90 did not affect SDCBP stability or expression (Supplementary Fig. S4e, f). On the contrary, the positive effect of SDCBP on HSP90 expression at the protein level (Fig. 4e, f) but not at the mRNA level (Supplementary Fig. S4g) prompted us to clarify whether SDCBP influences HSP90 protein stability. As shown in Fig. 4g and Supplementary Fig. S4h, overexpression of SDCBP stabilized the HSP90 protein by increasing its half-life via the ubiquitin–proteasome pathway, whereas knockdown of SDCBP greatly reduced HSP90 stability. Collectively, the above results suggest that SDCBP is a key driver of HSP90 Kbu.

SDCBP is an adapter protein containing two tandem PDZ domains^24,28^, and truncated mutants of SDCBP lacking the PDZ1 or PDZ2 domain, designated SDCBP-△PDZ1 and SDCBP-△PDZ2, respectively, were constructed to determine the motif responsible for the SDCBP-HSP90 interaction (Fig. 4h, upper panel). Wild-type SDCBP and SDCBP-△PDZ1 could precipitate HSP90, but we could not detect this interaction when the PDZ2 domain was deleted (Fig. 4h, lower panel). We also found that the PDZ2 domain alone precipitated HSP90 protein, suggesting that the PDZ2 motif was responsible for the SDCBP-HSP90 interaction (Supplementary Fig. S4i). To further identify the sites in HSP90 essential for its association with SDCBP, molecular docking (MD) was performed (Fig. 4i, upper panel). Three plasmids expressing different mutants of HSP90 (designated mut #1, mut #2, mut #3) were constructed, and the co-IP results indicated that amino acids 208–218 in HSP90 are required for its binding to SDCBP (Fig. 4i, lower panel). Since the amino acids 208–218 in HSP90 were also found to be critical for its interaction with HDAC11 (Fig. 4j), we next elucidated whether SDCBP interferes with HSP90-HDAC11 complex formation. In the presence of SDCBP, a significant reduction in HDAC11-bound HSP90 was observed, accompanied by an increase in SDCBP-bound HSP90 (Fig. 4k).

The role of the PDZ2 domain in SDCBP and the amino acids 208–218 in HSP90 in the regulation of HSP90 Kbu status was determined. PDZ2 alone significantly increased the Kbu level of HSP90 protein and SDCBP-△PDZ2 could not exert this effect (Fig. 4l). In addition, SDCBP failed to affect the Kbu status of HSP90-mut #3 protein in ESCC cells (Fig. 4m). These results suggest the important role of the PDZ2 domain and amino acids 208–218 in the function of SDCBP and HSP90, respectively, as well as in their interaction. The in vitro enzymatic reactions showed that both full-length SDCBP and PDZ2 motif alone could increase HSP90 Kbu level, but SDCBP-△PDZ2 could not (Supplementary Fig. S4j). Functionally, the PDZ2 motif was required for the role of SDCBP in increasing HSP90 expression and activation of the AKT signaling pathway (Supplementary Fig. S4k). Taken together, these data suggest that KAT8 acylates HSP90 at K754, and SDCBP competes with HDAC11 for binding to HSP90, leading to stabilization and Kbu modification of HSP90.

### HSP90 mediates the role of SDCBP in promoting 5-FU chemoresistance

**Fig. 5:**
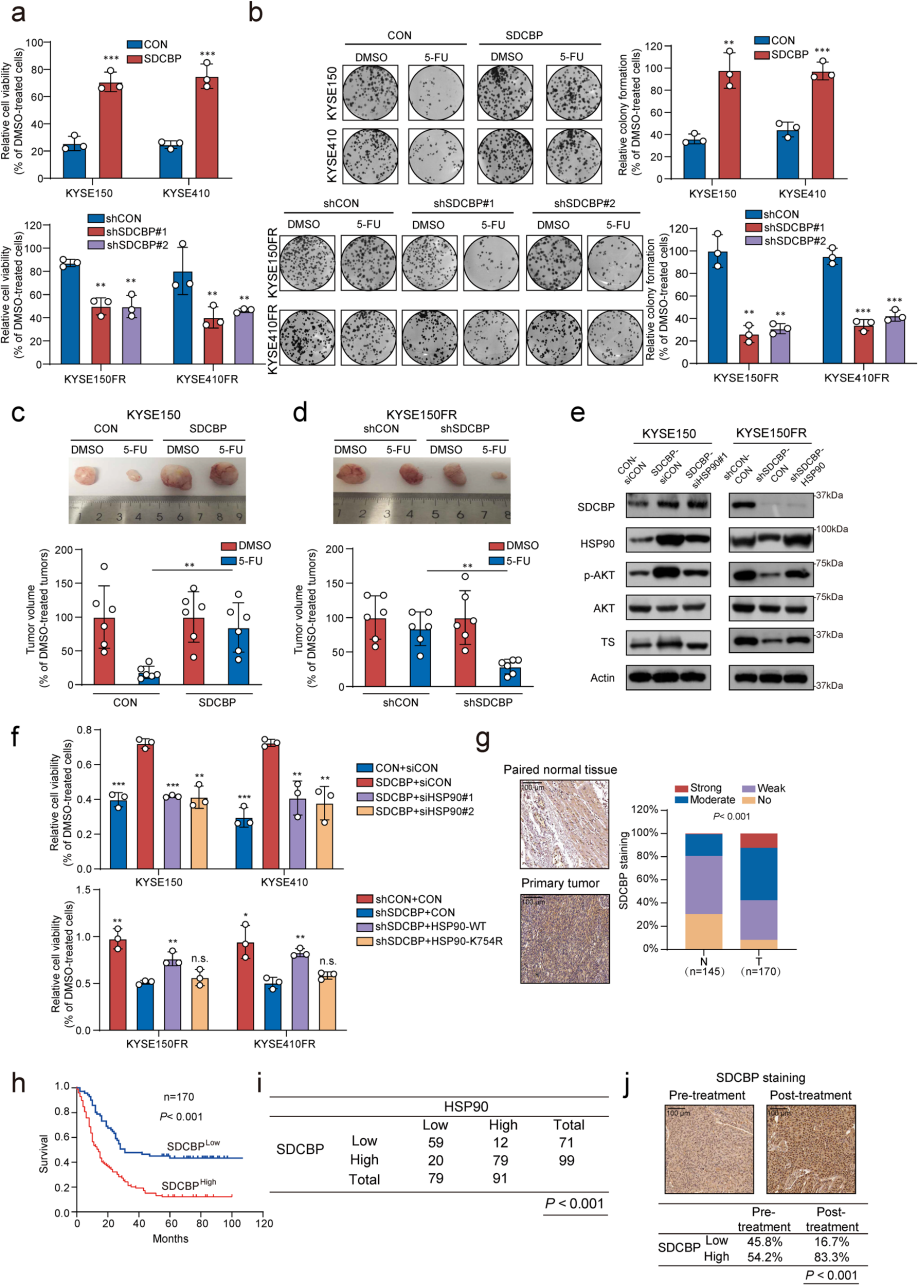
HSP90 mediates the role of SDCBP in promoting cancer chemoresistance. **a**, **b** Cell viability assay and colony formation assay showing the effect of SDCBP on the 5-FU sensitivity of ESCC cells. **c**, **d** Subcutaneous xenografts were established with SDCBP-overexpressing and SDCBP-knockdown cells, and the mice were treated with 5-FU or vehicle (*n* = 6). Representative images of tumors are shown, and tumor growth were quantified. Bars, SDs; *P < 0.05; **P < 0.01; ***P < 0.001. **e** Western blot analysis of SDCBP, HSP90, p-AKT, AKT and TS in the indicated ESCC cell lines. **f** Cell viability assay comparing 5-FU sensitivity in ESCC cells with manipulated SDCBP and/or HSP90 expression. **g** Expression pattern of SDCBP in a tumor tissue microarray consisting of 170 ESCC tissues and 145 normal tissues. **h** Kaplan–Meier analysis of overall survival for 170 ESCC patients stratified according to tumor SDCBP expression. **i** Correlation analysis between the expression of HSP90 and SDCBP. **j** Expression pattern of SDCBP in paired pre- and post-treatment samples from ESCC patients who received 5-FU-based neoadjuvant chemotherapy and subsequent surgery. Bars, SDs; *P < 0.05; **P < 0.01; ***P < 0.001.

The role of SDCBP in chemoresistance remains unclear. Gain- and loss-of-function experiments showed that overexpression of SDCBP reduced the sensitivity of cells to 5-FU treatment both in vitro and in vivo, whereas knockdown of SDCBP had the opposite effects (Fig. 5a–d). In addition, the increased 5-FU resistance, as well as AKT signaling pathway activation and high TS expression induced by SDCBP overexpression, were significantly abrogated by the knockdown of HSP90. In contrast, the knockdown of SDCBP enhanced 5-FU sensitivity in ESCC cells, and this effect was attenuated by overexpression of HSP90 (Fig. 5e, f). These results demonstrated that HSP90 is regulated by the SDCBP and plays a critical role in 5-FU resistance in ESCC.

We next sought to pinpoint the clinical relevance of SDCBP in ESCC; IHC analysis of SDCBP in the same tissue microarrays shown in Fig. 2 indicated that SDCBP expression was significantly higher in tumor tissues than in the paired normal tissues (Fig. 5g; Supplementary Table S2). Kaplan–Meier survival analysis showed that patients with high tumor SDCBP expression had significantly shorter survival times (median survival time = 14.0 months) than patients with low tumor SDCBP expression (median survival time = 31.0 months) (log-rank test, *P* < 0.001; Fig. 5h). Moreover, a positive correlation between HSP90 and SDCBP expression was demonstrated (Pearson *χ*^2^ test, *P* < 0.001) (Fig. 5i). In addition, analysis of paired pre- and post-treatment ESCC samples showed an elevated SDCBP level after 5-FU treatment (Fig. 5j), corroborating the important role of SDCBP in chemoresistance.

### Identification of V020-9974 as a lead compound targeting HSP90 Kbu to suppress chemoresistance

**Fig. 6:**
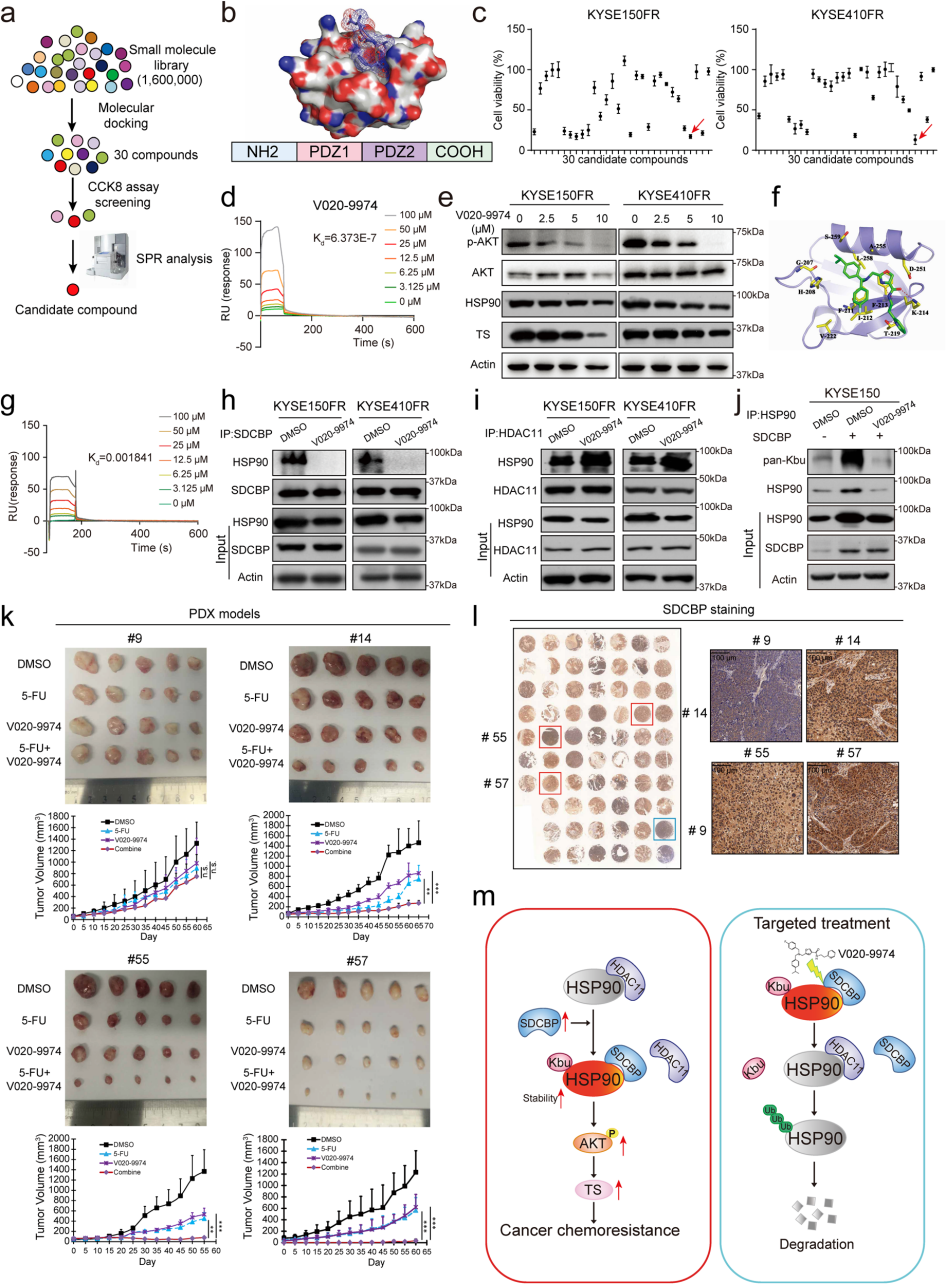
Identification of a lead compound targeting HSP90 Kbu to suppress chemoresistance. **a** Flow chart showing the strategy used to screen SDCBP inhibitors. **b** 3D structure of the SDCBP protein. **c** Comparison of the survival inhibitory activity of 30 candidate compounds. **d** Biacore analysis showing the binding between V020-9974 and the SDCBP protein. **e** Western blot analysis of p-AKT, AKT, HSP90 and TS in ESCC cells treated with different concentrations of V020-9974. **f** Predicted model of V020-9974 binding to the PDZ2 domain of SDCBP. **g** The mutant SDCBP protein was purified and subjected to Biacore analysis to evaluate its binding to V020-9974. **h**, **i** IP assay showing that V020-9974 disrupted the SDCBP-HSP90 interaction but enhanced the HDAC11-HSP90 interaction. **j** V020-9974 decreased Kbu modification of HSP90. **k** The establishment of the PDX models and the treatment strategy. Representative images are shown, and tumor growth was monitored. **l** Representative images showing the expression of SDCBP in PDX#9, #14, #55 and #57 (right panel). **m** Summary diagram. Bars, SDs; *P < 0.05; **P < 0.01; ***P < 0.001.

As demonstrated above, SDCBP can increase HSP90 Kbu by binding competitively to HDAC11, with the PDZ2 domain as the functional motif, which constitutes an ideal target for cancer therapy. High-throughput virtual screening was performed on a compound library consisting of 1.6 million small molecules, and the top 30 compounds were selected based on docking score ranking and cluster analysis (Fig. 6a, b; Supplementary Table S3). Among the 30 candidate compounds selected, three compounds—D332-0030, V020-9974 and V025-1375—inhibited cell survival and AKT signaling activation in ESCC cells (Fig. 6c; Supplementary Fig. S5a). In particular, V020-9974 had the highest binding affinity for SDCBP, as indicated by surface plasmon resonance (SPR) analysis (Fig. 6d; Supplementary Fig. S5b, c). Thus, we selected V020-9974 as our research focus. A series of in vitro and in vivo experimental assays showed that V020-9974 reduced the protein levels of p-AKT and TS and exerted anticancer effects in a dose-dependent and time-dependent manner without observed side effects (Fig. 6e; Supplementary Fig. S5d–i).

As shown in Fig. 6f, V020-9974 was predicted to anchor into the SDCBP cavity by forming bonds with phenylalanine (F) 213, K214 and threonine (T) 219, the amino acids within the PDZ2 domain. We thus purified a His-tagged SDCBP mutant (SDCBP^F213A/K214A/T219A^) and then subjected it to SPR analysis. As expected, V020-9974 exhibited a markedly lower binding affinity for the SDCBP mutant (1.8 mM) than for wild-type SDCBP (0.63 µM) (Fig. 6g), confirming that V020-9974 directly binds to the PDZ2 domain in the SDCBP protein, which is also the motif required for the SDCBP-HSP90 interaction. Combining these results with the finding that V020-9974 did not decrease SDCBP protein expression in ESCC cells (Supplementary Fig. S5j), we speculated that V020-9974 may interfere with the association between HSP90 and SDCBP. To support our hypothesis, we assessed the interaction between SDCBP and HSP90 in the presence of V020-9974. Immunoprecipitation analysis indicated that V020-9974 not only decreased the binding of HSP90 to SDCBP but also increased the HSP90-HDAC11 interaction, therefore reducing the Kbu modification and expression level of HSP90 in ESCC cells (Fig. 6h–j).

The bioactivity of V020-9974 in modulating chemoresistance was determined in vitro and in vivo. Cell Counting Kit-8 (CCK-8) and colony formation assays showed that V020-9974 significantly abolished 5-FU resistance in ESCC cells (Supplementary Fig. S6a, b). In addition, xenograft models were established, and markedly smaller tumors and lower Ki-67 proliferation indexes were observed in mice treated with the combination of 5-FU and V020-9974 than in mice treated with V020-9974 or 5-FU alone (Supplementary Fig. S6c, d). No significant toxicity was observed in the animals (Supplementary Fig. S6e). Previous studies reported that the PDZ1 inhibitor (PDZ1i) of SDCBP inhibited the invasion of glioblastoma and breast cancer cells^26,29^. Here, we also tested whether PDZ1i could increase 5-FU sensitivity. Our data indicated that PDZ1i treatment did not affect 5-FU resistance in ESCC cells (Supplementary Fig. S6f, g).

Furthermore, human ESCC PDX models were utilized to evaluate the therapeutic potential of V020-9974 in a preclinical setting. V020-9974 markedly enhanced 5-FU sensitivity in PDX#14, PDX#55 and PDX#57 but not in PDX#9 (Fig. 6k; Supplementary Fig. S6h). IHC staining of the 4 human ESCC specimens from which the PDX models were derived revealed high SDCBP expression in tumor#14, tumor#55 and tumor#57 and low SDCBP expression in tumor#9 (Fig. 6l). This difference may explain the different responses of the PDX models to V020-9974. These results show that the combination of V020-9974 and 5-FU may have good application prospects for the treatment of ESCC patients with high SDCBP expression.

## Discussion

During the last few decades, great advances have been made in understanding PTMs, which fundamentally impact cellular physiology and phenotypes in eukaryotic organisms^30,31^. Beyond histone acylation modifications, the discovery of nonhistone acylation modifications has opened a new avenue of research; however, the map of the biological functions and precise regulatory mechanisms is far from complete. In the present study, among a panel of lysine acylation modifications, only Kbu was found to be abnormally upregulated in chemoresistant cancer cells. Kbu was initially discovered as a normal straight chain butyrylation modification^32^ and was recently identified as a novel chemical mark in histones that regulates sperm cell differentiation^33^ and plant adaptation^34^. To date, the function of Kbu in cancer has not been reported. Here, we provide the first butyrylome profile in human cancer, discovering 334 proteins and 608 sites with Kbu modification, among which 105 proteins and 123 sites were differentially modified in 5-FU resistant cancer cells. More importantly, we report for the first time that Kbu functionally contributes to drug resistance and identified HSP90 as a nonhistone substrate for Kbu modification.

HSP90 is an important molecular chaperone protein that regulates the stability and activity of more than 300 client proteins, many of which are involved in cancer progression. Several types of PTMs, e.g., phosphorylation, acetylation and SUMOylation, have been reported to regulate HSP90 activity and fine-tune it to the needs of the client proteins and the cell^35^. Here, we provide the first evidence that Kbu modification of HSP90 enhances its protein stability to drive chemoresistance in cancer cells. PTMs are dynamic and reversible modifications^36^, and identification of the corresponding writers and erasers is essential for deciphering the regulatory mechanisms^37^. Although two acetyltransferases, CBP and P300, were recently found to catalyze Kbu modification of histones^8^, the enzymes responsible for Kbu deposition or removal in nonhistone proteins are unknown. We reveal for the first time that KAT8 is the writer while HDAC11 is the eraser of HSP90 Kbu. The possibility that other writers or erasers also exist cannot be excluded, and determining how these enzymes cooperate to exert exquisite regulation, therefore, warrants further detailed investigation.

Esophageal cancer is the sixth most common cause of cancer-related deaths worldwide, and ESCC is the predominant histologic subtype in Asia^37,38^. However, relapsed ESCC always acquires resistance to chemotherapy and is often inoperable. Although recent studies on the development of HSP90 inhibitors are encouraging, none of these inhibitors have yet been approved by the FDA for cancer therapy. Therefore, targeting the HSP90 interaction network was proposed to be a potential optimized strategy^39^. The present study delineates the mechanisms by which SDCBP acts as the upstream regulator of HSP90; specifically, SDCBP increases the Kbu modification and expression level of HSP90 by binding competitively to HDAC11. SDCBP has been reported to be involved in a diverse array of functions, including the trafficking of transmembrane proteins, immunomodulation, exosome biogenesis, and tumorigenesis^40^. We therefore sought to discover small molecules that could target SDCBP and establish a therapeutic strategy to overcome chemoresistance. In a recent study, a small molecule inhibitor of the PDZ1 domain, PDZ1i, was identified based on the PDZ1 domain as a potential anti-prostate cancer agent^41^. Here, based instead on the PDZ2 domain, V020-9974 was identified and demonstrated to target the SDCBP-HSP90 interaction and thus suppress HSP90 Kbu and decrease HSP90 protein stability, subsequently sensitizing ESCC cells to 5-FU treatment in multiple cell and mouse models, providing a potential therapeutic strategy for advanced ESCC.

In conclusion, our study demonstrates that K754 butyrylation of HSP90 contributes significantly to 5-FU resistance. Mechanistically, via competitive binding to HDAC11, SDCBP regulates the Kbu modification and stability of HSP90. Pharmacological inhibition of the SDCBP-HSP90 interaction with a lead compound offers a potential strategy to overcome chemoresistance (Fig. 6m). Our findings not only open a new chapter in Kbu research but also provide a theoretical basis for drug development.

## Materials and methods

### Sample preparation

5-FU-resistant cell line KYSE150-FR and the parental cells KYSE150 were used to perform the butyrylome. Protein sample preparation and LC–MS/MS using tandem mass tag (TMT) labeling were performed at PTM Biolab Co. Ltd. (Hangzhou, Zhejiang, China). Cells were lysed, and the protein concentration was determined. For PTM experiments, inhibitors were also added to the lysis buffer, e.g., 3 μM trichostatin A (TSA) and 50 mM nicotinamide (NAM) to inhibit butyrylation. For digestion, proteins in solution were reduced with 5 mM dithiothreitol for 30 min at 56 °C and alkylated with 11 mM iodoacetamide for 15 min at room temperature in the dark. The protein sample was then diluted by adding 100 mM TEAB to obtain a urea concentration of less than 2 M. Finally, trypsin was added at a 1:50 trypsin-to-protein mass ratio for the first digestion overnight and a 1:100 trypsin-to-protein mass ratio for a second 4 h digestion.

### TMT labeling

After trypsin digestion, peptides were desalted on a Strata X C18 SPE column (Phenomenex, Torrance, CA, USA) and vacuum-dried. Peptides were reconstituted in 0.5 M TEAB and processed according to the TMT kit manufacturer’s protocol. In brief, one unit of TMT reagent was thawed and reconstituted in acetonitrile. The peptide mixtures were then incubated for 2 h at room temperature and pooled, desalted and dried by vacuum centrifugation.

### Pan-antibody-based PTM enrichment

To enrich modified peptides, tryptic peptides dissolved in NETN buffer (100 mM NaCl, 1 mM EDTA, 50 mM Tris-HCl, 0.5% NP-40; pH 8.0) were incubated with prewashed antibody beads (PTM Biolab Co. Ltd.) at 4 °C overnight with gentle shaking. Then, the beads were washed four times with NETN buffer and twice with H_2_O. The bound peptides were eluted from the beads with 0.1% trifluoroacetic acid. Finally, the eluted fractions were combined and vacuum-dried. For LC–MS/MS analysis, the resulting peptides were desalted with C18 ZipTips (Millipore, Bedford, MA, USA) according to the manufacturer’s instructions.

### LC–MS/MS analysis

Tryptic peptides were dissolved in solvent A (0.1% formic acid and 2% acetonitrile in water) and directly loaded onto a ReproSil-Pur Basic C18 analytical column (1.9 μm, 100 μm × 25 cm). Peptides were separated with a gradient of solvent B (0.1% formic acid in 90% acetonitrile) increasing from 9% to 25% over 24 min, increasing from 25% to 35% over 8 min, increasing to 80% over 4 min and remaining at 80% for the last 4 min on an EASY-nLC 1000 UPLC system (Thermo Fisher Scientific, Waltham, MA, USA) at a constant flow rate of 400 nL/min. The separated peptides were analyzed in a Q ExactiveTM HF-X mass spectrometer (Thermo Fisher Scientific) with a nanoelectrospray ion source. The electrospray voltage applied was 2.0 kV. The full MS scan resolution was set to 120,000 over a scan range of 350–1600 m/z. The 20 most abundant precursors were then selected for further MS/MS analyses with 10 s dynamic exclusion duration. High-energy collision dissociation (HCD) fragmentation was performed at a normalized collision energy (NCE) of 28%. Fragment ions were detected in the Orbitrap at a resolution of 20,000. The fixed first mass was set at 100 m/z. The automatic gain control (AGC) target was set at 1E5, with a maximum injection time of 100 ms.

### Cell lines and reagents

The human esophageal squamous cell carcinoma (ESCC) cell lines KYSE150 and KYSE410 obtained from DSMZ (Braunschweig, Germany), as well as the fluorouracil (5-FU)-resistant sublines KYSE150-FR and KYSE410-FR that we established^12^, were maintained in RPMI-1640 medium (Thermo Fisher Scientific) supplemented with 10% fetal bovine serum (FBS; Invitrogen, Gaithersburg, MD, USA) in a humidified atmosphere of 5% CO_2_ at 37 °C. All cell lines were authenticated by short tandem repeat profiling and tested negative for Mycoplasma contamination. 5-FU was purchased from Selleck Chemicals (Houston, TX, USA), and the small molecule compounds were obtained from TargetMol (Boston, MA, USA). Butyryl-Coenzyme A was obtained from Cayman Chemical (Ann Arbor, Michigan, USA).

### ESCC tissues, microarray analysis and IHC analysis

IHC analysis was performed on paraffin-embedded tumor sections as previously described^42^. Pre- and post-treatment tumor specimens from ESCC patients who received neoadjuvant chemotherapy and subsequent surgery were collected at the First Affiliated Hospital, Zhengzhou University. Tissue microarrays containing 170 samples of ESCC and 145 samples of nontumor tissue (Shanghai Outdo Biotech, Shanghai, China) were used to analyze the expression of HSP90 and SDCBP (Proteintech, Chicago, IL, USA). The staining intensity was scored as follows: no staining, 0; weak staining, 1; moderate staining, 2; strong staining, 3. Samples with a score of 0 or 1 were classified as having low expression, while those with a score of 2 or 3 were classified as having high expression.

### Plasmids, transfection, infection and gene knockout by CRISPR/Cas9 genome editing

The HSP90, SDCBP, SDCBP-PDZ2, SDCBP-△PDZ1, SDCBP-△PDZ2, SDCBP-△PDZ1 + 2, HDAC11 and KAT8 overexpression plasmids, the short hairpin RNAs (shRNAs) against SDCBP were obtained from TranSheep Bio (Shanghai, China). The siRNAs against HSP90, HDAC11, KAT8 and SDCBP were obtained from TsingKe Biotech (Beijing, China). The annealed sgRNA oligos were inserted into the lentiCRISPRv2 vector (Addgene plasmid 52961)^43^, which was a gift from Feng Zhang (Massachusetts Institute of Technology Cambridge, MA, USA), to generate the HSP90 knockout plasmids. Transfection and infection were performed using Lipofectamine 3000 reagent (Thermo Fisher Scientific) according to the manufacturer’s instructions as previously described^42^. The sgRNA, shRNA and siRNA sequences are listed in Supplementary Table S4. All mutants were generated using a pair of oligonucleotide primers designed with mismatched nucleotides (Supplementary Dataset S4).

### Cell viability assay

Cell viability was determined using a Cell Counting Kit-8 (CCK-8; Dojindo Molecular Technologies Inc., Rockville, MD, USA) as previously described^44^. According to the instructions, cells in 96-well plates were exposed to various concentrations of drugs and incubated with CCK-8 solution for 2 h at 37 °C. Cell viability was quantified by measuring the absorbance of the solution at 450 nm.

### Colony formation assay

The colony formation assay was performed as previously described^45^. Cells were seeded into 6-well plates at a density of 2000 cells per well and cultured for 14 days. The cells were fixed with 75% ethanol and stained with 0.2% crystal violet, and the colonies were counted.

### Quantitative real-time PCR (qRT–PCR)

Total RNA was isolated with TRIzol reagent (Thermo Fisher Scientific) and reverse transcribed with a PrimeScript II First-Strand cDNA Synthesis Kit (Takara, Dalian, China). qRT–PCR was performed according to the instructions using SYBR Green qPCR SuperMix (TransGen Biotech, Beijing, China). GAPDH was used as the internal control. The primers are listed in Supplementary Dataset S4.

### Western blot analysis

Cell lysates were collected, and western blot analysis was performed as previously described^46^. The primary antibodies used included pan-antibodies against a panel of lysine acylation modifications (PTM Biolab Co. Ltd., Hangzhou, Zhejiang, China), as well as antibodies against HSP90, SDCBP, HDAC11, KAT8, TS, Myc, HA (Proteintech), p-AKT and AKT (Cell Signaling Technology, Beverly, MA, USA), Flag (Sigma–Aldrich, St. Louis, MO, USA), HDAC11 and actin (Santa Cruz Biotechnology, Santa Cruz, CA, USA). The antibodies are listed in Supplementary Table S5.

### IP-MS and Co-IP

Protein digestion and MS analysis were executed as previously described^47^. In brief, proteins were digested with trypsin, vacuum-freeze-dried, and resuspended in anhydrous acetonitrile solution, then desalted with MonoTIPTM C18 Pipette Tip (GL Sciences, Tokyo, Japan). Peptide samples were analyzed with an Orbitrap Fusion Lumos mass spectrometer (Thermo Fisher Scientific). Then raw data were analyzed using Proteome Discoverer (Thermo Fisher Scientific) and Spectronaut (Omicsolution Co., Ltd., Shanghai, China) software. Protein and peptide FDRs were set to 1%. For Co-IP, cell lysates were incubated with IgG (Santa Cruz Biotechnology) and protein A/G Sepharose beads (Santa Cruz Biotechnology) at 4 °C for 1 h. The supernatant was mixed with the appropriate primary antibody overnight at 4 °C prior to a 4 h incubation with protein A/G Sepharose beads. After washing with PBS and lysis buffer, the beads were mixed with 5× SDS/PAGE loading buffer for Western blot analysis.

### Protein purification and surface plasmon resonance (SPR) analysis

The pET-28b vector was used to construct the plasmids pET-28b-SDCBP, pET-28b-SDCBP^F213A/K214A/T219A^, pET-28b-SDCBP-PDZ2, pET-28b-SDCBP-△PDZ2, pET-28b-HDAC11, pET-28b-HDAC11-H143A, pET-28b-KAT8, pET-28b-KAT8-K274R expressing wild-type and mutant histidine (His)-tagged fusion proteins. The pGEX-6P-1 vector was used to construct the plasmids pGEX-6P-1-HSP90 expressing wild-type glutathione S-transferase (GST)-tagged HSP90 fusion proteins. The plasmids were transformed into *E. coli* (BL21), and 0.5 mM isopropyl β-D-thiogalactopyranoside (IPTG) was added for 2 h to induce SDCBP-His protein expression when the optical density of the bacterial culture at 600 nm was approximately 0.6. The bacteria were lysed by sonication, and the His-tagged SDCBP fusion protein was isolated by Ni-NTA affinity chromatography (Invitrogen) and analyzed by western blotting. While GST-tagged HSP90 fusion protein was purified by GSH (Beyotime Biotechnology, Shanghai, China) and analyzed by western blotting. SPR analysis was performed on a Biacore X100 system (GE Healthcare Life Sciences, Marlborough, MA, USA) as described previously^47^. In brief, recombinant SDCBP protein was dissolved in PBS and immobilized on a CM7 chip (GE Healthcare Life Science), which was pre-equilibrated with PBS containing 0.4‰ P20 according to the manufacturer’s instructions. Different concentrations of the SDCBP inhibitor (100, 50, 25, 12.5, 6.25, 3.125, and 0 μM) dissolved in running buffer were injected into the channel and analyzed at 25 °C. Binding events were recorded, and the binding curve and *K*_*d*_ values of the small molecule and the protein were obtained.

### GST pull-down assay

GST or HSP90-GST protein was incubated with Glutathione-Sepharose beads (Cytiva Life Sciences, Marlborough, MA, USA) for 2 h at 4 °C. The complexes were then resuspended with lysis buffer, and His-tagged proteins were added to the solution, and the mixture was incubated overnight at 4 °C. After washing, the pulled-down proteins were analyzed by SDS-PAGE and detected by western blot assay.

### MD

The nuclear magnetic resonance (NMR) structures of SDCBP (PDB ID: 1W9Q) and HSP90 (PDB ID: 4BQG) were downloaded from the RCSB Protein Data Bank. The docking model of the two proteins was generated by using Discovery Studio 4.5 (Accelrys Inc., San Diego, CA, USA). Protein docking was performed based on the ZDOCK score to determine the complex structures of SDCBP and HSP90. After optimization by RDOCK, the model was used for molecular dynamics simulations in the CHARMM27 force field with a 500-step steepest descent minimization followed by conjugate gradient minimization until convergence to 0.01 kcal/mol. Finally, the 100-ns MD simulations were performed with the GROMACS 5.1.2 software package.

### Pharmacophore modeling and high-throughput virtual screening

To construct a three-dimensional pharmacophore model for SDCBP–substrate interactions, the Surflex-Dock module in SYBYL-X2.0 (Tripos Associates Inc., St. Louis, MO, USA) was used for high-throughput virtual screening of a compound database consisting of 1.6 million small molecules (ChemDiv, San Diego, CA, USA). The crystal structure of the PDZ2 domain of SDCBP in complex with an interleukin 5 receptor alpha peptide (PDB ID: 1OBX) was obtained from the Protein Data Bank (PDB)^48,49^. During the preparation of the receptor SDCBP, the space where the interleukin 5 receptor alpha peptide was placed was selected as the active pocket via the Protomol module in SYBYL-X2.0, and all water molecules were removed. The molecules with a docking score in the top 1% were subjected to a second screening using the default docking parameters. The compounds with favorable characteristics were purchased for the subsequent biological activity assay.

### In vitro butyrylation assay

Protein butyrylation assay was carried out as described^8^. The reaction mixture contained 50 mM Tris (pH 7.9), 10% glycerol, 1 mM DTT, 10 mM sodium butyrate, 10 μM butyryl-CoA, 2.5 μg of highly purified substrate (HSP90) and/or 20–100 ng of the enzyme protein. The mixture was incubated at 30 °C for 1 h, and the Kbu level was detected by western blot assay.

### Tumor xenograft assay

All animal experiments were approved by the Experimental Animal Ethics Committee of Guangzhou Medical University and performed according to institutional guidelines. Xenograft tumors were generated by subcutaneously injecting 1 × 10^6^ ESCC cells into the flanks of nude mice aged 6–8 weeks as previously described^50^. Mice were fed a standard laboratory chow diet and water ad libitum. When the subcutaneous tumors reached ~5 mm in diameter, the mice were intraperitoneally injected twice weekly with 5-FU (20 mg/kg) and the inhibitor (5 mg/kg) alone or in combination. Tumor size was monitored with calipers every three days, and tumor volume was calculated using the equation V = (length × width^2^)/2. All measured tumor sizes were normalized to the vehicle-treated group.

### Hematologic analysis

Alanine aminotransferase (ALT) and aspartate aminotransferase (AST) in mouse serum were measured using kits from HuiLi Biotech Ltd. (Changchun, China). Hemoglobin (HGB) and blood cells from mice, including lymphocytes, neutrophils, platelets (PLTs), red blood cells (RBCs) and white blood cells (WBCs), were analyzed with a fully automatic hematology analyzer (BC-2800Vet, Mondrary, Shenzhen, China).

### Statistical analysis

All in vitro experiments were repeated at least three times. The data are expressed as the means ± SD and were compared using ANOVA. The expression levels of SDCBP and HSP90 in tumor samples were compared with those in nontumor tissues using paired or unpaired *t*-tests. The associations among the expression levels of SDCBP and HSP90 were determined using the Pearson rank correlation coefficient. Survival analysis was performed by the Kaplan–Meier method with the log-rank test. *P* < 0.05 was considered significant.

### Study approval

All animal experiments were approved by the Experimental Animal Ethics Committee of Guangzhou Medical University, and mice were cared for under standard conditions according to institutional guidelines. The human ESCC specimens were collected in accordance with the Declaration of Helsinki and were approved by the Ethics Committee of the First Affiliated Hospital of Zhengzhou University (No. SS-2020-003). Informed consent was obtained from each participant.

## Supplementary information

Supplementary Figures and Tables

Dataset S1

Dataset S2

Dataset S3

Dataset S4

## Data Availability

All data needed to evaluate the conclusions in the article are present in the article and/or the Supplementary materials. The datasets used and/or analyzed during the current study are available from the corresponding author upon reasonable request.
