# Supplementary material for: Lysine butyrylation of HSP90 regulated by KAT8 and HDAC11 confers chemoresistance
Source: Cell Discov. 2023 Jul 18;9:74. doi: 10.1038/s41421-023-00570-y (PMC10352258; doi:10.1038/s41421-023-00570-y)
Supplement: Supplementary file 1 — Supplementary Figures and Tables [file 41421_2023_570_MOESM1_ESM.pdf]

Supplementary Materials for

Lysine butyrylation of HSP90 regulated by KAT8 and HDAC11 confers chemoresistance

Yan He *et al.*

*Corresponding author. Email: lib2128@163.com (Bin Li).

This PDF file includes:

Figures S1 to S6

Tables S1 to S5

Other Supplementary Materials for this manuscript include the following:

Datasets S1 to S4

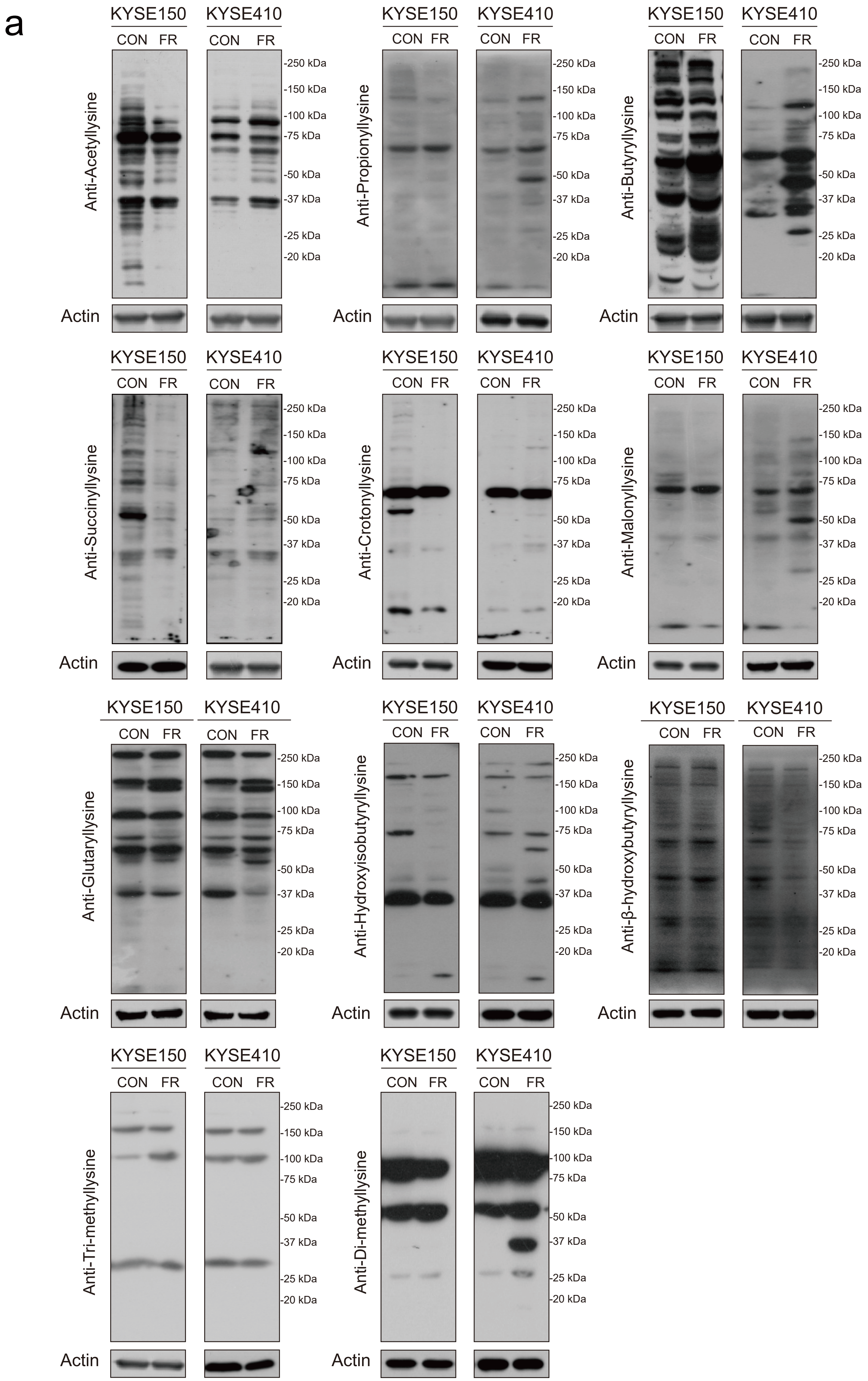
a
KYSE150
KYSE410
CON
FR
Anti-Acetyllysine
Anti-Propionyllysine
Anti-Butyryllysine
Anti-Succinyllysine
Anti-Crotonyllysine
Anti-Malonyllysine
Anti-Glutaryllysine
Anti-Hydroxyisobutyryllysine
Anti-β-hydroxybutyryllysine
Anti-Tri-methyllysine
Anti-Di-methyllysine
Actin
-250 kDa
-150 kDa
-100 kDa
-75 kDa
-50 kDa
-37 kDa
-25 kDa
-20 kDa

Fig. S1

Fig. S1. (a) Comparison of the profiles of the 9 newly identified lysine acylation modifications, as well as the trimethyllysine and dimethyllysine modifications, in 5-FU-resistant cells and parental cells revealed that the level of only Kbu was significantly increased in resistant ESCC cells.

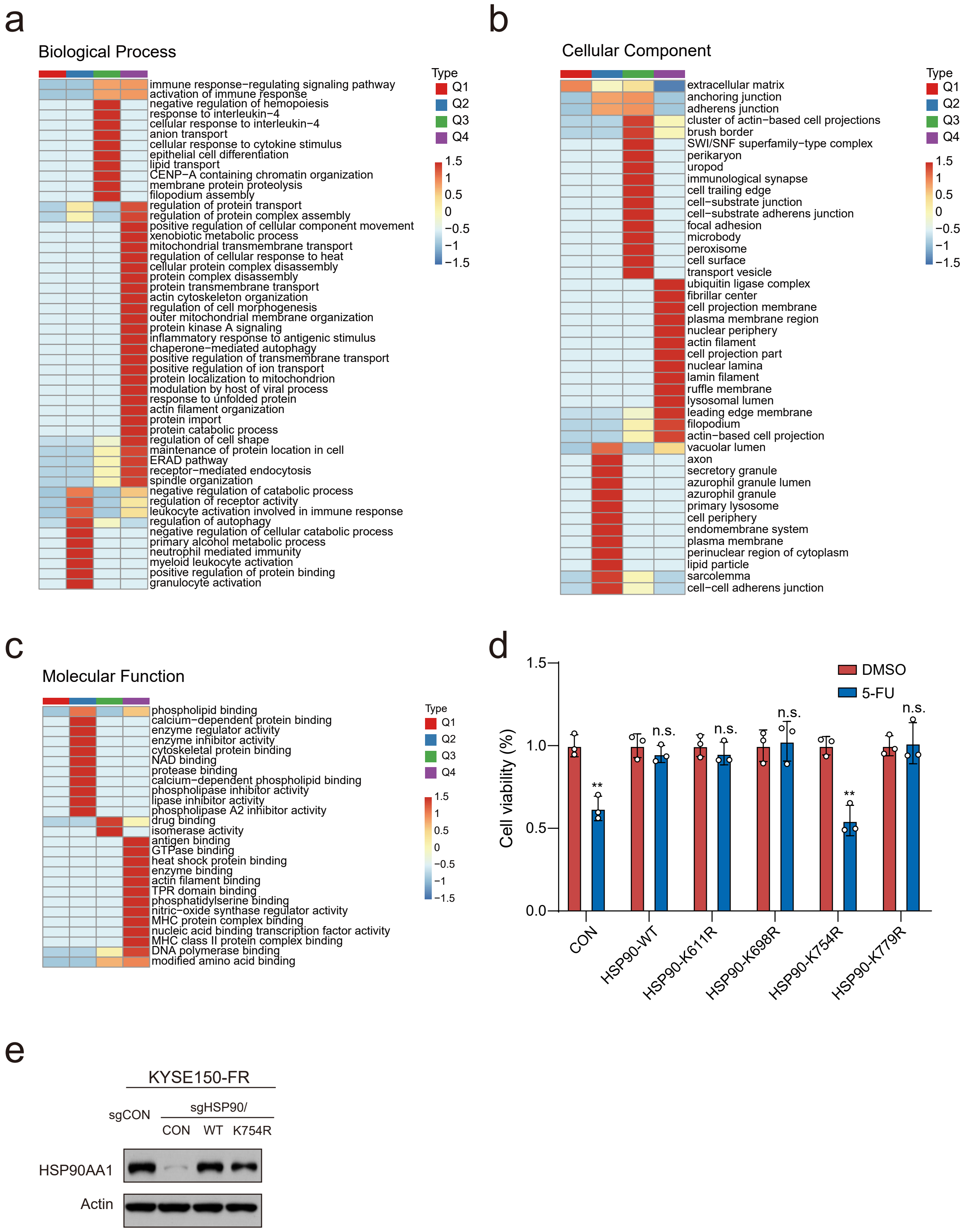
a
Biological Process
Type
Q1
Q2
Q3
Q4
1.5
1
0.5
0
−0.5
−1
−1.5
immune response−regulating signaling pathway
activation of immune response
negative regulation of hemopoiesis
response to interleukin−4
cellular response to interleukin−4
anion transport
cellular response to cytokine stimulus
epithelial cell differentiation
lipid transport
CENP−A containing chromatin organization
membrane protein proteolysis
filopodium assembly
regulation of protein transport
regulation of protein complex assembly
positive regulation of cellular component movement
xenobiotic metabolic process
mitochondrial transmembrane transport
regulation of cellular response to heat
cellular protein complex disassembly
protein complex disassembly
protein transmembrane transport
actin cytoskeleton organization
regulation of cell morphogenesis
outer mitochondrial membrane organization
protein kinase A signaling
inflammatory response to antigenic stimulus
chaperone−mediated autophagy
positive regulation of transmembrane transport
positive regulation of ion transport
protein localization to mitochondrion
modulation by host of viral process
response to unfolded protein
actin filament organization
protein import
protein catabolic process
regulation of cell shape
maintenance of protein location in cell
ERAD pathway
receptor−mediated endocytosis
spindle organization
negative regulation of catabolic process
regulation of receptor activity
leukocyte activation involved in immune response
regulation of autophagy
negative regulation of cellular catabolic process
primary alcohol metabolic process
neutrophil mediated immunity
myeloid leukocyte activation
positive regulation of protein binding
granulocyte activation
b
Cellular Component
Type
Q1
Q2
Q3
Q4
1.5
1
0.5
0
−0.5
−1
−1.5
extracellular matrix
anchoring junction
adherens junction
cluster of actin−based cell projections
brush border
SWI/SNF superfamily−type complex
perikaryon
uropod
immunological synapse
cell trailing edge
cell−substrate junction
cell−substrate adherens junction
focal adhesion
microbody
peroxisome
cell surface
transport vesicle
ubiquitin ligase complex
fibrillar center
cell projection membrane
plasma membrane region
nuclear periphery
actin filament
cell projection part
nuclear lamina
lamin filament
ruffle membrane
lysosomal lumen
leading edge membrane
filopodium
actin−based cell projection
vacuolar lumen
axon
secretory granule
azurophil granule lumen
azurophil granule
primary lysosome
cell periphery
endomembrane system
plasma membrane
perinuclear region of cytoplasm
lipid particle
sarcolemma
cell−cell adherens junction
c
Molecular Function
Type
Q1
Q2
Q3
Q4
1.5
1
0.5
0
−0.5
−1
−1.5
phospholipid binding
calcium−dependent protein binding
enzyme regulator activity
enzyme inhibitor activity
cytoskeletal protein binding
NAD binding
protease binding
calcium−dependent phospholipid binding
phospholipase inhibitor activity
lipase inhibitor activity
phospholipase A2 inhibitor activity
drug binding
isomerase activity
antigen binding
GTPase binding
heat shock protein binding
enzyme binding
actin filament binding
TPR domain binding
phosphatidylserine binding
nitric−oxide synthase regulator activity
MHC protein complex binding
nucleic acid binding transcription factor activity
MHC class II protein complex binding
DNA polymerase binding
modified amino acid binding
d
DMSO
5-FU
Cell viability (%)
1.5
1.0
0.5
0.0
**
n.s.
n.s.
n.s.
**
n.s.
CON
HSP90-WT
HSP90-K611R
HSP90-K698R
HSP90-K754R
HSP90-K779R
e
KYSE150-FR
sgCON
sgHSP90/
CON
WT
K754R
HSP90AA1
Actin

Fig. S2

Fig. S2. (a-c) GO enrichment analysis showing that Kbu may be functionally involved in various biological processes and pathways. **(d)** Cell viability assay showing that Kbu of HSP90 at K754 contributed to 5-FU resistance in ESCC cells. The mutation of lysine to arginine (R) mimicked the debutyrylated state. **(e)** Overexpression of HSP90-WT or HSP90-K754R in HSP90-deficient ESCC cells. Bars, SDs; *, $P < 0.05$; **, $P < 0.01$; ***, $P < 0.001$.

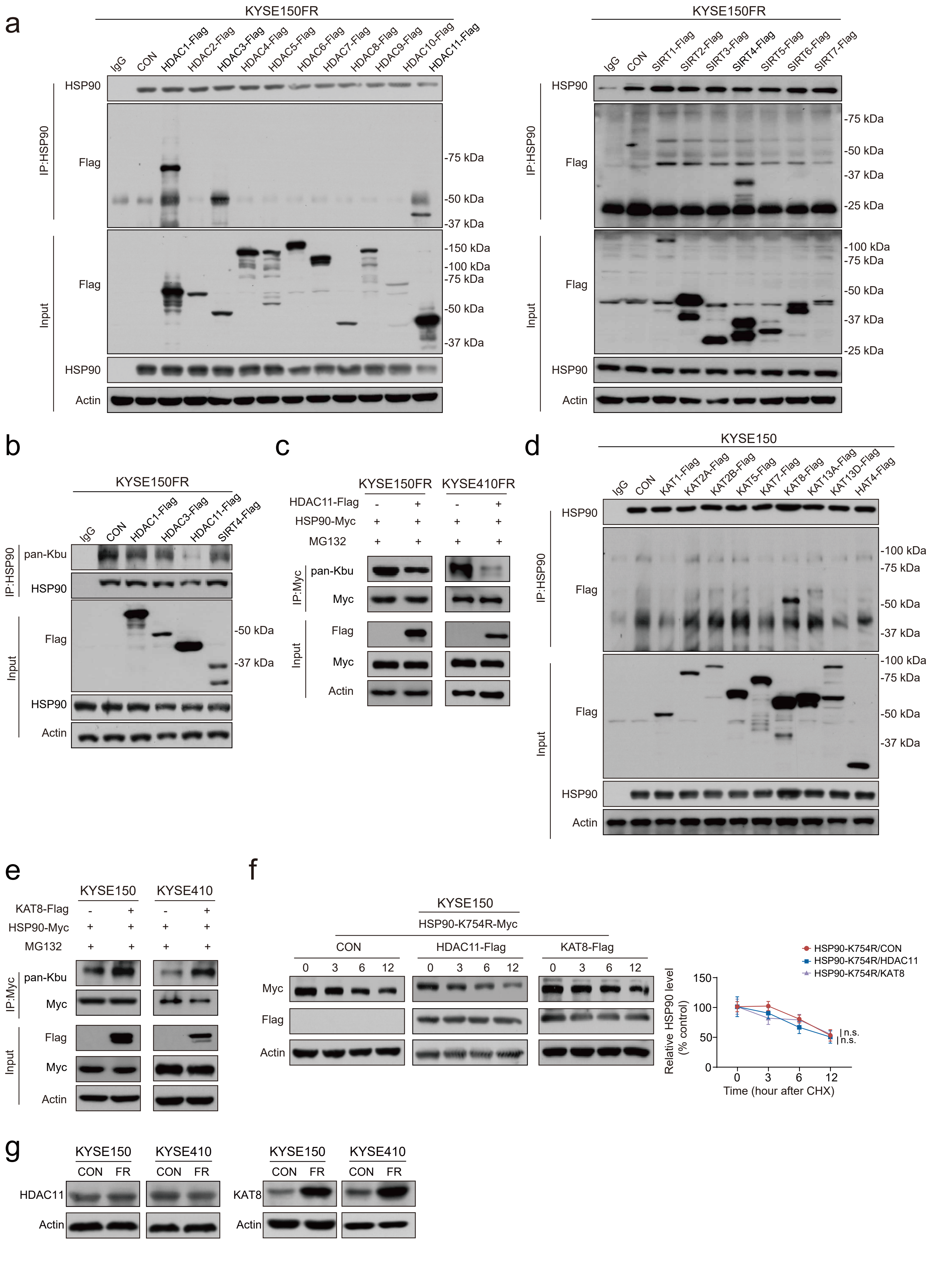
a
KYSE150FR
IgG
CON
HDAC1-Flag
HDAC2-Flag
HDAC3-Flag
HDAC4-Flag
HDAC5-Flag
HDAC6-Flag
HDAC7-Flag
HDAC8-Flag
HDAC9-Flag
HDAC10-Flag
HDAC11-Flag
IP:HSP90
Input
HSP90
Flag
Actin
-75 kDa
-50 kDa
-37 kDa
-150 kDa
-100 kDa
SIRT1-Flag
SIRT2-Flag
SIRT3-Flag
SIRT4-Flag
SIRT5-Flag
SIRT6-Flag
SIRT7-Flag
-25 kDa
b
pan-Kbu
SIRT4-Flag
c
KYSE410FR
HDAC11-Flag
HSP90-Myc
MG132
IP:Myc
Myc
d
KYSE150
KAT1-Flag
KAT2A-Flag
KAT2B-Flag
KAT5-Flag
KAT7-Flag
KAT8-Flag
KAT13A-Flag
KAT13D-Flag
HAT4-Flag
e
KYSE410
KAT8-Flag
f
HSP90-K754R-Myc
0
3
6
12
HSP90-K754R/CON
HSP90-K754R/HDAC11
HSP90-K754R/KAT8
Relative HSP90 level (% control)
150
100
50
Time (hour after CHX)
n.s.
g
FR
HDAC11
KAT8

Fig. S3

Fig. S3. (a) A series of HDAC or SIRT expression plasmids were transfected into ESCC cells as indicated, and the cells were then subjected to immunoprecipitation with an anti-HSP90 antibody. **(b)** HDAC11 but not HDAC1, HDAC3 or SIRT4 decreased Kbu modification of HSP90. **(c)** Effect of HDAC11 on HSP90 Kbu levels in the presence of proteasome inhibitor MG132. Cells were treated with MG132 (10 μM) for 8 hours before harvest. **(d)** ESCC cells were transfected with a series of HATs and then subjected to an immunoprecipitation assay. **(e)** Effect of KAT8 on HSP90 Kbu levels in the presence of proteasome inhibitor MG132. Cells were treated with MG132 (10 μM) for 8 hours before harvest. **(f)** Analysis of protein stability after CHX treatment in the ESCC cells overexpressing HSP90-K754R. **(g)** Expression levels of HDAC11 and KAT8 in 5-FU-resistant/sensitive cells. Bars, SDs; *, $P < 0.05$; **, $P < 0.01$; ***, $P < 0.001$.

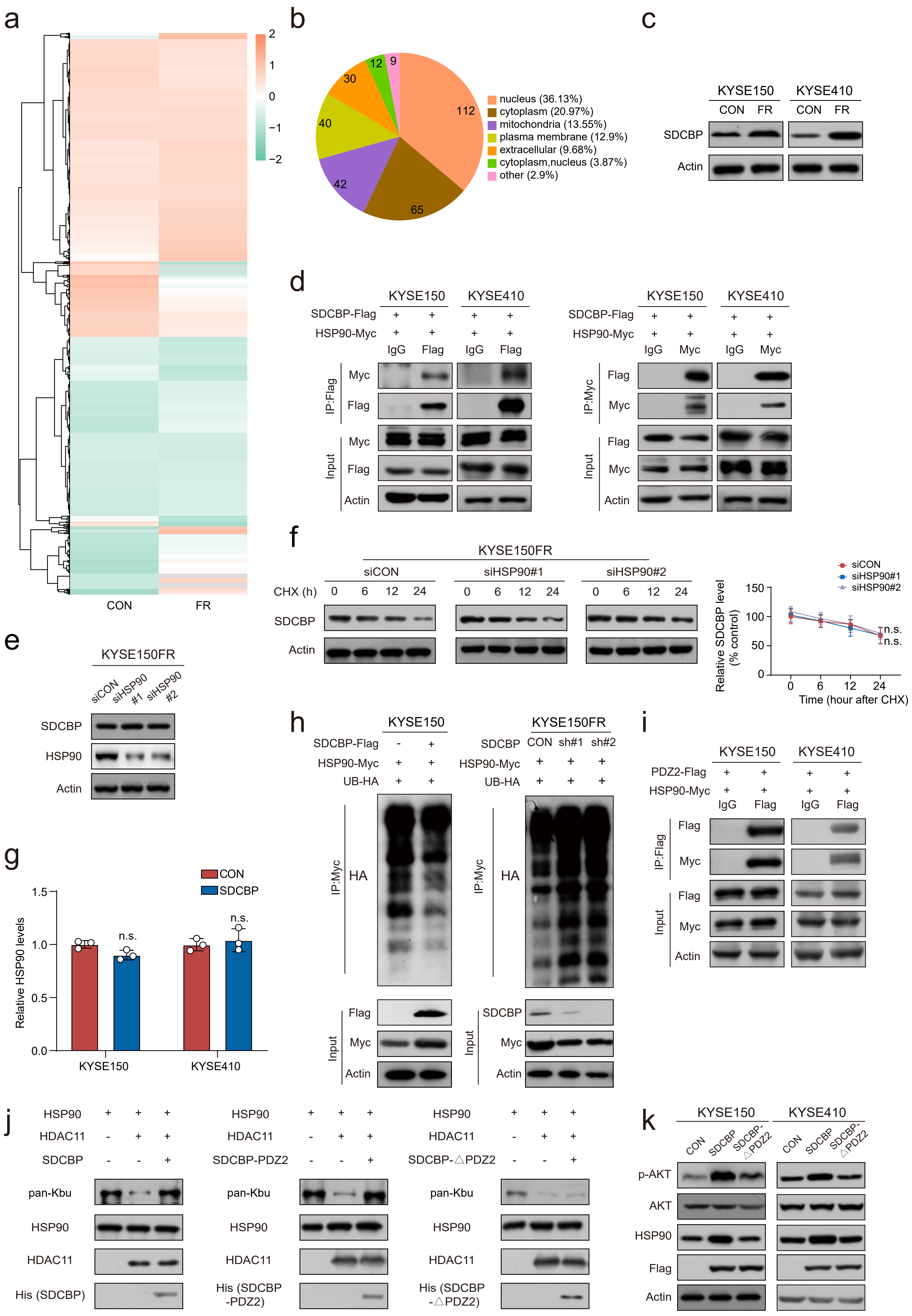
a
CON
FR
2
1
0
−1
−2
b
112
65
42
40
30
12
9
nucleus (36.13%)
cytoplasm (20.97%)
mitochondria (13.55%)
plasma membrane (12.9%)
extracellular (9.68%)
cytoplasm,nucleus (3.87%)
other (2.9%)
c
KYSE150
KYSE410
CON FR CON FR
SDCBP
Actin
d
KYSE150
KYSE410
SDCBP-Flag
HSP90-Myc
IgG Flag IgG Flag
IP:Flag
Myc
Flag
Input
Myc
Flag
Actin
IgG Myc IgG Myc
IP:Myc
Input
e
KYSE150FR
siCON
siHSP90 #1
siHSP90 #2
SDCBP
HSP90
Actin
f
KYSE150FR
siCON
siHSP90#1
siHSP90#2
CHX (h)
0 6 12 24
SDCBP
Actin
siCON
siHSP90#1
siHSP90#2
Relative SDCBP level (% control)
Time (hour after CHX)
n.s.
n.s.
g
CON
SDCBP
Relative HSP90 levels
n.s.
n.s.
KYSE150
KYSE410
h
KYSE150
KYSE150FR
SDCBP-Flag
HSP90-Myc
UB-HA
SDCBP
CON sh#1 sh#2
IP:Myc
HA
Input
Flag
Myc
Actin
SDCBP
i
KYSE150
KYSE410
PDZ2-Flag
HSP90-Myc
IgG Flag IgG Flag
IP:Flag
Flag
Myc
Input
Actin
j
HSP90
HDAC11
SDCBP
SDCBP-PDZ2
SDCBP-△PDZ2
pan-Kbu
HSP90
HDAC11
His (SDCBP)
His (SDCBP -PDZ2)
His (SDCBP -△PDZ2)
k
KYSE150
KYSE410
CON
SDCBP
SDCBP-△PDZ2
p-AKT
AKT
HSP90
Flag
Actin

Fig. S4

Fig. S4. (a) Heatmap showing DEPs in 5-FU resistant cells. **(b)** Subcellular distribution of the DEPs. **(c)** Expression of SDCBP in paired 5-FU-resistant/sensitive ESCC cells. **(d)** Immunoprecipitation assay showing the interaction between exogenous SDCBP and HSP90. **(e)** Effect of HSP90 knockdown on SDCBP expression in ESCC cells. **(f)** Analysis of SDCBP protein stability after CHX treatment in the HSP90-knockdown ESCC cells. **(g)** The mRNA level of HSP90 was determined in SDCBP-overexpressing ESCC cells. **(h)** Effect of SDCBP on the ubiquitination of HSP90. **(i)** The interaction between HSP90 and SDCBP-PDZ2 was determined by immunoprecipitation. **(j)** Comparative butyrylation of HSP90. HSP90 and HDAC11 was incubated in the presence or absence of PDZ2. **(k)** Effects of SDCBP and SDCBP-△PDZ2 on HSP90 expression and PI3K/AKT activation. Bars, SDs; *, $P < 0.05$; **, $P < 0.01$; ***, $P < 0.001$.

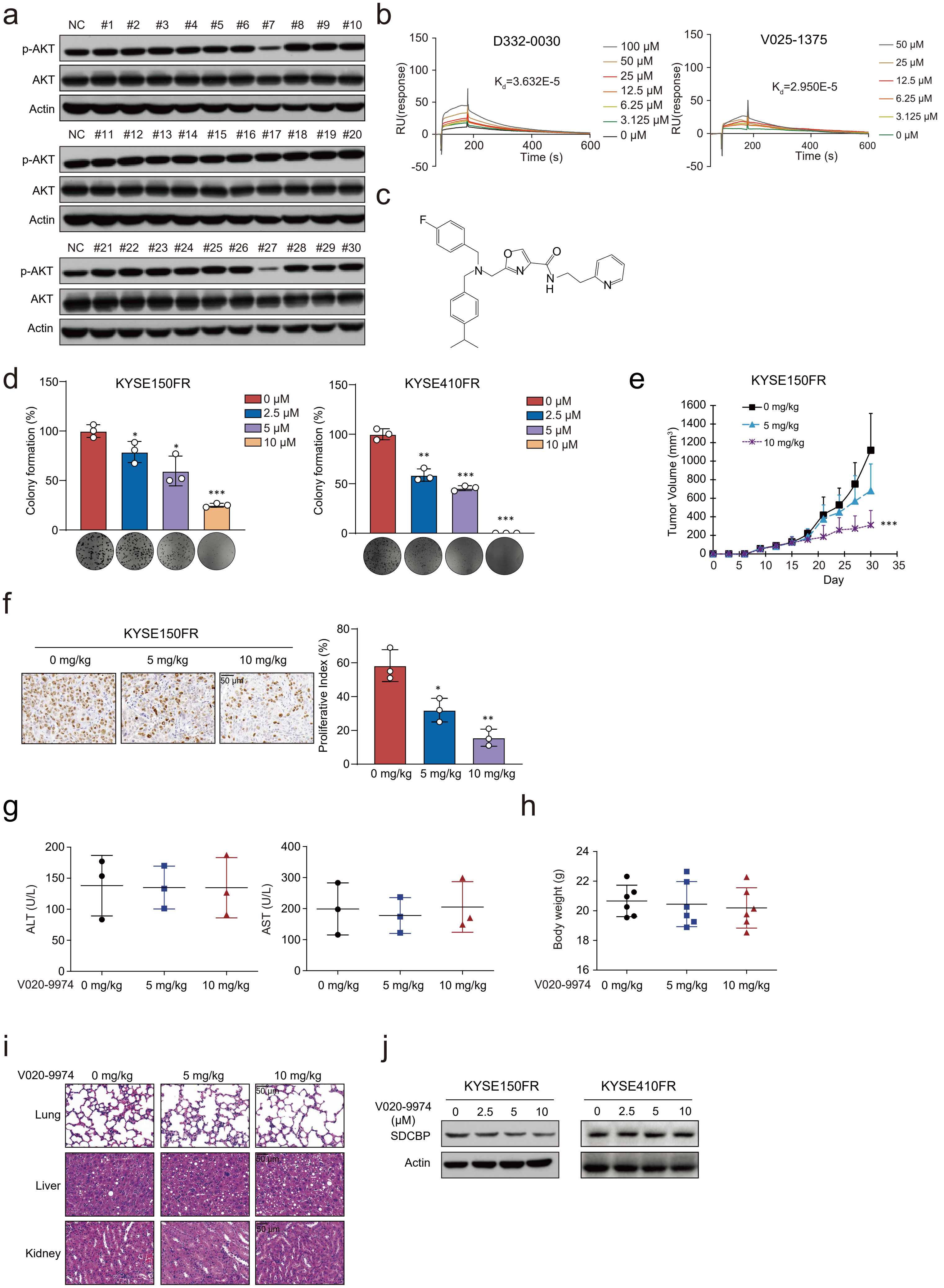
a
NC #1 #2 #3 #4 #5 #6 #7 #8 #9 #10
p-AKT
AKT
Actin
NC #11 #12 #13 #14 #15 #16 #17 #18 #19 #20
NC #21 #22 #23 #24 #25 #26 #27 #28 #29 #30
b
D332-0030
K_d=3.632E-5
V025-1375
K_d=2.950E-5
RU(response)
Time (s)
c
d
KYSE150FR
KYSE410FR
Colony formation (%)
0 μM
2.5 μM
5 μM
10 μM
e
KYSE150FR
0 mg/kg
5 mg/kg
10 mg/kg
Tumor Volume (mm³)
Day
f
KYSE150FR
Proliferative Index (%)
g
ALT (U/L)
AST (U/L)
V020-9974
h
Body weight (g)
i
Lung
Liver
Kidney
j
KYSE150FR
KYSE410FR
V020-9974 (μM)
SDCBP
Actin

Fig. S5

Fig. S5. (a) Western blot showing the levels of p-AKT and AKT in cells treated with 30 candidate inhibitors. **(b)** Biacore analysis indicating the binding between the SDCBP protein and compounds D332-0030 and V025-1375. **(c)** Chemical structure of V020-9974. **(d)** Effects of V020-9974 on the colony formation abilities. **(e-f)** Effects of V020-9974 on the growth and proliferation (as evaluated by the Ki-67 proliferation index) of ESCC tumor xenografts. **(g)** ALT and AST levels in mice were monitored. **(h)** Body weights of the mice in the treatment and control groups. **(i)** H&E staining of lung, liver and kidney specimens collected from mice in the treatment and control groups. **(j)** Effect of V020-9974 on SDCBP protein expression in ESCC cell lines. Bars, SDs; *, $P < 0.05$; **, $P < 0.01$; ***, $P < 0.001$.

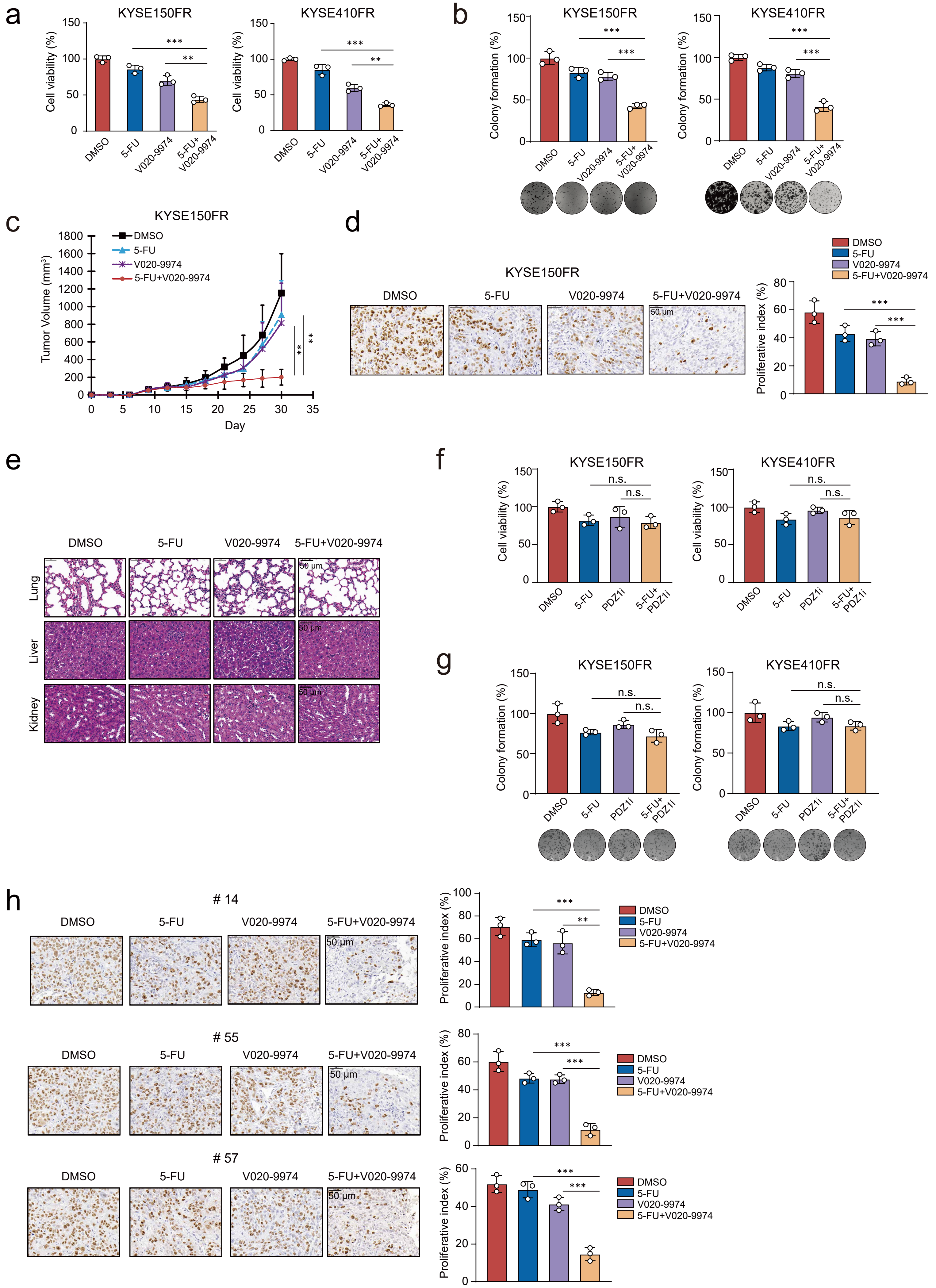
a
KYSE150FR
KYSE410FR
Cell viability (%)
DMSO
5-FU
V020-9974
5-FU+ V020-9974
b
Colony formation (%)
c
KYSE150FR
Tumor Volume (mm³)
Day
5-FU+V020-9974
d
Proliferative index (%)
50 μm
e
Lung
Liver
Kidney
f
PDZ1i
5-FU+ PDZ1i
n.s.
g
h
14
55
57

Fig. S6

Fig. S6. **(a)** The cell viability assay showed that V020-9974 overcame 5-FU resistance in 5-FU-resistant cells. **(b)** Colony formation assay showing that V020-9974 increased the sensitivity of ESCC cells to 5-FU. **(c-d)** The animal experiment and tumor growth curves showing the growth of ESCC xenografts treated with 5-FU and V020-9974 alone or in combination (c) and the Ki-67 proliferation index as determined by IHC staining (d). **(e)** H&E staining of organs collected from mice in the treatment and control groups. **(f-g)** Detection of the therapeutic efficiency of PDZ1i in 5-FU-resistant cells by cell viability assay (f) and colony formation assay (g). **(h)** In the presence of 5-FU, V020-9974 significantly reduced the Ki-67 proliferation index in PDX#14, PDX#55 and PDX#57 but not in PDX#9. Bars, SDs; *, $P < 0.05$; **, $P < 0.01$; ***, $P < 0.001$.

Table S1. Correlation between HSP90 expression levels and clinicopathological parameters in 170 patients with esophageal cancer.

Variable	n	Low HSP90	High HSP90	*P* value
Age (years)				
≤55	25	10	15	
>55	145	69	76	0.4824
Gender				
Female	39	21	18	
Male	131	58	73	0.2987
T-Stage				
1/2	40	31	9	
3/4	130	48	82	*P* <0.001
N-Stage				
N0	79	43	36	
N1	89	35	54	0.050
Grade				
I & II	132	59	73	
III & IV	38	20	18	0.3875
Stage				
1 & 2	79	24	55	
3 & 4	82	49	33	*P* <0.001

Table S2. Correlation between SDCBP expression levels and clinicopathological parameters in 170 patients with esophageal cancer.

Variable	n	Low SDCBP	High SDCBP	*P* value
Age (years)				
≤55	25	8	17	
>55	145	63	82	0.2837
Gender				
Female	39	19	20	
Male	131	52	79	0.3178
T-Stage				
1/2	40	27	13	
3/4	130	44	86	*P* <0.001
N-Stage				
N0	79	37	42	
N1	89	33	56	0.2004
Grade				
I & II	132	49	83	
III & IV	38	22	16	0.0221
Stage				
1 & 2	79	26	53	
3 & 4	82	43	39	0.0123

Table S3. The 30 candidate compounds.

	Name	Total_Score	MW	PSA	PBC	CLOGP	CLOGP_ERROR
1	E002_2569	8.52	504.5344	150.361	9	3.1313	0
2	V023_7190	8.34	500.5855	107.535	10	5.5501	0
3	V011_8667	8.28	500.6534	169.493	10	2.4701	0
4	F343_0572	8.28	464.5783	154.667	9	0.5005	0
5	E002_2419	8.27	503.5215	92.472	7	4.7367	0
6	C561_3100	8.19	436.5929	104.008	10	4.8043	0
7	C528_0777	8.11	479.5466	136.982	10	0.626	41
8	G678_0797	7.94	467.559	133.563	7	3.3082	40
9	E750_0131	7.94	467.5573	106.984	11	4.7927	20
10	G305_2163	7.9	419.4085	239.666	6	1.2677	0
11	C562_1581	7.86	514.637	150.212	10	3.0432	30
12	C200_6700	7.8	490.594	108.308	9	3.5404	10
13	V017_6620	7.78	487.547	127.746	10	3.1541	0
14	F796_0141	7.77	364.4409	116.627	5	3.702	0
15	D332_0030	7.76	436.5466	59.175	8	4.6008	0
16	D264_0838	7.74	445.5103	91.9	5	3.8991	0
17	D674_0070	7.74	415.4412	93.524	7	4.0328	0
18	V020_9974	7.73	486.5804	72.869	11	5.074	42
19	Y041_8051	7.71	438.4548	152.907	9	-0.0428	0
20	D510_0986	7.7	469.5747	64.721	7	5.2179	10
21	F255_0231	7.7	468.5469	111.514	8	4.7491	0
22	E984_1311	7.67	409.5245	78.593	8	1.8225	0
23	C667_0368	7.66	377.4778	122.954	9	1.4395	40
24	F796_0164	7.65	429.5142	104.865	5	2.853	0
25	F714_0940	7.63	473.585	117.162	10	3.367	41
26	E902_0156	7.61	474.5548	124.884	7	1.9076	0
27	G545_0489	7.6	490.5691	91.881	8	5.576	30
28	E570_2828	7.6	497.6495	98.752	8	4.0632	0
29	E848_0422	7.59	393.5004	90.788	8	2.669	0
30	V025_1375	7.58	494.6489	97.683	9	5.4939	41

Table S4. The target sequences of sgRNA, shRNA and siRNA.

Name	Sequence
sgHSP90#1	TTCTCTTGCAGGTGAACCTA
sgHSP90#2	GGTTGAGACGTTCGCCTTTC
shSDCBP#1	GAGAAGATTACCATGACCATT
shSDCBP#2	GTACTTCAGATCAATGGTGAA
siHSP90#1	AACCCTGACCATTCCATTATT
siHSP90#2	GUUUGAGAACCUCUGCAAA
siHDAC11#1	GAGACUUCAUGGACGACAATT
siHDAC11#2	CACACGAGGCGCUAUCUUATT
siKAT8#1	CGAUCACCAAGGUGAAGUA
siKAT8#2	GCAAGCAUGAUGAGAUCAA
siSDCBP#1	GACUCUUAAGAUUAUGUAATT
siSDCBP#2	AGAAUGUCAUUGGAUUGAATT

Table S5. The list of indicated antibodies.

Antibody	Cat No.	Manufacturer
HSP90	13171-1-AP	Proteintech
Butyryllysine	PTM-301	Jingjie PTM Biolabs
HDAC11	67949-1-Ig	Proteintech
HDAC11	sc-390737	Santa Cruz
HER2	4290P	Cell Signaling Technology
p53	10442-1-AP	Proteintech
Androgen Receptor	22089-1-AP	Proteintech
FKBP52	10655-1-AP	Proteintech
HSP70	10995-1-AP	Proteintech
SDCBP	22399-1-AP	Proteintech
Ubiquitin	PTM-1106	Jingjie PTM Biolabs
KAT8	13842-1-AP	Proteintech
β-actin	sc-47778	Santa Cruz
p-AKT	4060S	Cell Signaling Technology
AKT	4691S	Cell Signaling Technology
TS	15047-1-AP	Proteintech
His	66005-1-Ig	Proteintech
Flag	F1804	Sigma Aldrich
Myc	60003-2	Proteintech
HA	51064-2-AP	Proteintech
acetyllysine	PTM-105	Jingjie PTM Biolabs
2-Hydroxyisobutyryllysine	PTM-802	Jingjie PTM Biolabs
Propionyllysine	PTM-201	Jingjie PTM Biolabs
Succinyllysine	PTM-401	Jingjie PTM Biolabs
Crotonyllysine	PTM-501	Jingjie PTM Biolabs
Tri-methyllysine	PTM-601	Jingjie PTM Biolabs
Di-methyllysine	PTM-606	Jingjie PTM Biolabs
Malonyllysine	PTM-901	Jingjie PTM Biolabs
Glutaryllysine	PTM-1151	Jingjie PTM Biolabs
β-hydroxybutyryllysine	PTM-1201	Jingjie PTM Biolabs

Dataset S1 (separate file)

Cell Kbu-proteome dataset

Dataset S2 (separate file)

Cell proteome dataset

Dataset S3 (separate file)

The interacting proteins were identified from immunoprecipitation coupled with liquid chromatography tandem mass spectrometry (IP-MS)

Dataset S4 (separate file)

Primers
